# Role of Feeding and Novel Ripening System to Enhance the Quality and Production Sustainability of Curd Buffalo Cheeses

**DOI:** 10.3390/foods12040704

**Published:** 2023-02-06

**Authors:** Marika Di Paolo, Valeria Vuoso, Rosa Luisa Ambrosio, Anna Balestrieri, Giovanna Bifulco, Aniello Anastasio, Raffaele Marrone

**Affiliations:** 1 Department of Veterinary Medicine and Animal Production, University of Naples Federico II, 80137 Naples, Italy; 2 Istituto Zooprofilattico Sperimentale del Mezzogiorno, 80055 Portici, Italy

**Keywords:** dry ricotta cheeses, semi-hard cheeses, accelerated ripening, fatty acids profile, food safety

## Abstract

The buffalo dairy sector is extending its boundaries to include new buffalo cheese productions beyond mozzarella, overcoming some barriers that make cheeses expensive and unsustainable. This study aimed to evaluate the effects of both the inclusion of green feed in the diet of Italian Mediterranean buffaloes and an innovative ripening system on buffalo cheese quality, providing solutions capable of guaranteeing the production of nutritionally competitive and sustainable products. For this purpose, chemical, rheological, and microbiological analyses were carried out on cheeses. Buffaloes were fed with or without the inclusion of green forage. Their milk was used to produce dry ricotta and semi-hard cheeses, ripened according to both respective traditional (MT) and innovative methods (MI); these are based on automatic adjustments of climatic recipe guided by the continuous control of pH. Green feed enhances the nutritional profile of the final products (high content of MUFAs and PUFAs). As far as the ripening method is concerned, to our knowledge, this is the first study that tests aging chambers, commonly used for meat, for the maturing of buffalo cheeses. Results pointed out the MI validity also in this field of application, as it shortens the ripening period without negatively compromising any of desirable physicochemical properties and the safety and hygiene of the final products. Conclusively, this research highlights the benefits of diets rich in green forage on productions and provides support for the ripening optimization of buffalo semi-hard cheeses.

## 1. Introduction

Dairy water buffalo (*Bubalus bubalis*) farming is a traditional Italian enterprise recently engaged in the intensification of rearing techniques [1]. Buffalo milk contributes 15% to total milk production in the world [2] and buffalo cheese production is growing in countries where most of the milk production comes from buffaloes [3]. Buffalo milk is used almost exclusively for cheese-making of mozzarella [4], a typical fresh and stringy-textured cheese certified with European Protected Designation of Origin (PDO) as “Mozzarella di Bufala Campana” (EC 103/2008). It is known that the chemical composition of buffalo milk gives the cheese excellent qualitative features, allowing the production of various value-added dairy products. The fatty acid profile as well as the microelements, calcium and vitamin A content of the buffalo milk offer excellent opportunities to produce cheeses with noble nutritional benefits [5]. To this end, Salzano et al. [6] demonstrated how the addition of green feed (30% of diet) to the buffalo diet could improve the quality of milk, significantly increasing the levels of health promoting biomolecules (L-carnitine and short-chain acylcarnitines, propionyl-L-carnitine, and δ-valerobetaine, etc.) and the percentage of beneficial long-chain polyunsaturated (PUFA) fatty acids.

Differently from the mozzarella cheese, various buffalo dairy products undergo a period of ripening whose duration varies according to the chosen cheese type. As far as the cured cheeses are concerned, both the quality of the milk and the choice of the ripening method play a crucial role in the final properties of end-products. A close relationship between the microbiota of raw milk and final products has been widely demonstrated [7]. Likewise, the peculiar properties of any ripened cheese are strictly dependent on the maturation processes since relative humidity, temperature and flow parameters are the main responsible for the changes in microbiological, organoleptic, and physical-chemical profiles [8]. Changes in pH and activity water in dairy products, following the ripening, could affect the viability of specific microorganisms, including spoilage and pathogenic bacteria that could contaminate the matrices along the entire food-chain [9]. Moreover, in the case of buffalo milk, it is worth highlighting the influence of its chemical properties on the ripening process. Specifically, Batool et al. [10] demonstrated how the size of fat globules as such as high buffering capacity and excessive syneresis slow down the development of acid, which is responsible for the rapid activation of biochemical processes (glycolysis, lipolysis and proteolysis) in the cheese [11,12]. Consequently, the ripening of buffalo cheese requires a fairly long period which results in a greater economic burden for food business operators.

Several authors have analyzed alternative climatic conditions to shorten the ripening time of cheese, preserving the role of ripening as tool capable to obtain the desired sensorial properties and reduce the concentration of undesired microorganisms [13,14]. In the last decade, some innovative ripening strategies have been built on the consolidated knowledge of the key role of matrix pH as one of the main factors influencing the rate at which biochemical reactions, which occur in food during maturation, proceed [11]. In particular, maturation cabinets have been designed with an integrated system for the instantaneous recognition of food deterioration through a continuous analysis of the pH; this continuous monitoring has the aim of guiding the automatic adjustment of the climatic parameters, reporting the pH values within a safe range and preserving the safety and the typical characteristics of foods. In this scenario, taking into account the economic and nutritional importance of buffalo milk, novel or existing ripening technologies should be explored to enhance the economic sustainability for manufacture of buffalo hard/semi-hard cheese, by acting on the ripening times. The reason why stakeholders and scientists come together for the discovery of systems/technologies that accelerate the ripening process could be summed up in the desire to enhance a buffalo supply chain that remains niche due to the costs associated with long production. The launch on the market of an accelerated ripening system can facilitate opening new horizons for cured buffalo cheeses, reducing the costs of production and the energy expenditure of the ripening cabinets. According to our knowledge, to date the market offers very few solutions to contain this problem, and the creation and application of new strategies would also allow us to know and analyze the potential disadvantages deriving from the use of these new technologies.

The aim of this work was to evaluate the effect of experimental ripening climatic recipes on two different buffalo dairy products. Particularly, the ripening tests were carried out in an industrial ripening plant by comparing the novel technologies with those traditionally adopted. Furthermore, to improve the milk quality and its content in fatty acids (FAs) and other nutrients [15,16], the cheeses were produced using the milk of buffaloes fed with different diets [17]. Through chemical-physical and microbiological analyses, the effects of the inclusion of green feed in the diet of Italian Mediterranean buffaloes and of the innovative ripening system on the quality of cheeses were evaluated.

## 2. Materials and Methods

### 2.1. Animals and Diets

The study was carried out in a commercial buffalo dairy farm (Verdi Praterie, Kr, Italy) located in southern Italy. Italian Mediterranean dairy buffaloes were maintained in pens with an allocation space of 16 m^2^ for buffalo and were milked twice a day in the morning and afternoon. Buffaloes were randomly divided into two groups: with (fresh group, FRS; *n* = 25) or without (control group, CTL; *n* = 25) the inclusion of green feed. The two diets were isonitrogenous and isoenergetic and differed mainly in the inclusion of green feed in FRS buffaloes. The amount of the diets is reported in Table S1.

### 2.2. Cheese Making and Ripening Process

**Figure 1:**
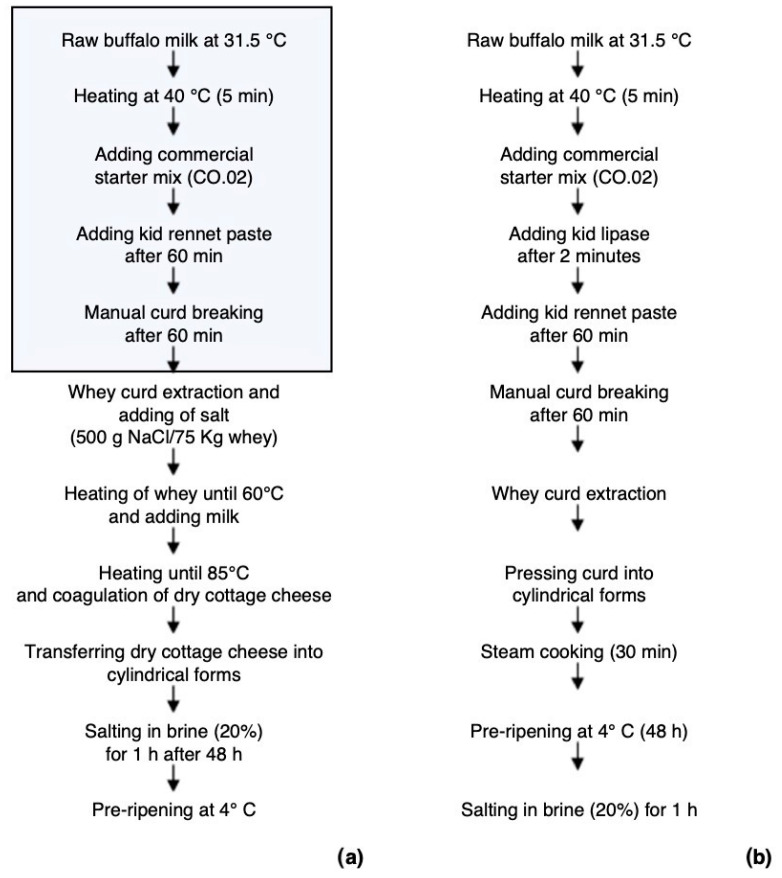
Flow diagrams of buffalo cheeses production of dry ricotta cheese (**a**) and semi-hard cheese (**b**).

The experimentation had the objective of testing two types of aged cheese produced with milk coming from both the control and treated buffalo groups. From July to September 2020, two batches of raw milk from each buffalo group (CTL and FRS) were collected in three different moments (three replications for each experimental group), and used to produce dry ricotta and semi-hard cheeses in a local dairy industry in southern Italy (Caseificio Bufà, Crotone, KR, Italy). Flow diagrams of buffalo cheeses production are shown in Figure 1. For both cheese-making processes, the raw milk was heated for 5 min at 40° and coagulated with a mixture of commercial starter (*Lactococcus lactis* spp. *cremoris*, *Lactococcus lactis* spp. *lactis*, *Lactococcus lactis* spp. *lactis* biovar. *diacetylactis*, SACCO SRL, CO, Italy) and rennet paste (75% chymosin, 25% pepsin; Caglificio Clerici S.p.a., Cadorago, Como, Italy) in 60 min. For semi-hard cheese making, the curd was cut into medium-size grains and, after extracting the whey, pressed into cylindrical shapes, and steamed at 100 °C for 30 min. After this phase, cheeses were refrigerated at 4°C for 48 h and then soaked in brine (salt, 20% *w*/*v*) for 1 h. For dry ricotta cheese making, the whey collected after the production of buffalo cheeses was mixed with 10% of milk (*v*/*v*) which was previously heated to 40 °C. The salt was then added (500 g NaCl/75 kg) and the solution heated up to 85 °C. Following coagulation, the cheese was transferred into cylindrical forms, soaked in brine (salt, 20% *w*/*v*) for 1 h and then refrigerated at 4 °C for 48 h.

Two different ripening conditions were carried out for each type of cured buffalo cheese (Table 1). For cheese ripened with the innovative method, the fundamental phases of the process (such as the draining, the stewing, the firming, the drying and ripening steps) have been carried out at controlled temperatures, relative humidity, and airflow. During ripening, cheeses were turned over once a week to keep their composition uniform. Twelve productions were made for each cheese: six cheeses were ripened in traditional method conditions (MT, long ripening time), and the other six cheeses were ripened in innovative method conditions (MI, short ripening time) into the Stagionello—European Patented Device and controlled pH—n. EP 2769276B1 (Figure S1). This method facilitated the monitoring of the physical and chemical state of cheese during the ripening by continuously measuring of the pH and climatic parameters (temperature, air flow, relative humidity—RH). This device had an internal control system that set these parameters and a monitoring system that recorded these variables. The innovation of the system does not lie only in the climatic conditions chosen, but in the ability of this technology to follow and detect in real time the process parameters of cheeses and modulate it by modifying the climatic parameters. Obviously, the six cheeses employed for testing each ripening condition included three cheeses made with the milk of buffalo belonging to the fresh group (inclusion of green feed, FRS) and three with milk of buffalo of the control group (without any inclusion of green feed, CTL).

### 2.3. Sampling Procedure

**Figure 2:**
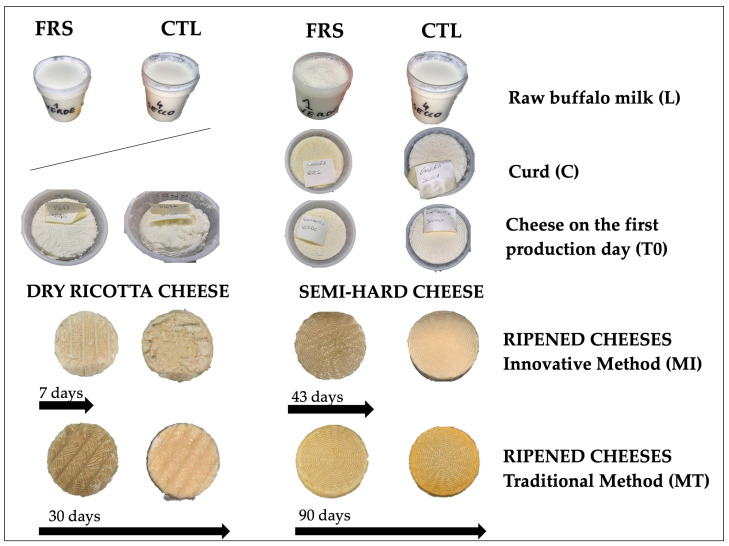
Experimental design schematization. FRS, production line with milk from buffaloes fed with feed rich in green fodder; CTL, production line with milk from buffaloes fed with feed without green fodder.

On each cheese making day, three samples of bulk milk from each vat were collected just before cheese-making, packed in 500 mL plastic flasks, and sent to the laboratory under refrigeration conditions (refrigerated boxes) for chemical and microbiological analyses. The experimental design is schematized in Figure 2. For each cheese-making session, a total of 30 and 24 dairy products for semi-hard cheese and dry ricotta cheese were collected, respectively. They consisted of 6 raw milk samples (L-FRS and L-CTL), 12 or 6 semi-finished products (n. 6 curd of semi-hard cheese, C-FRS and C-CTL; n. 6 cheese on the first production day, T0-FRS and T0-CTL), and 12 ripened cheeses (MI-FRS and MI-CTL; MT-FRS and MT-CTL). All samples were sent to the laboratory in refrigerated boxes and immediately analyzed for the determination of physicochemical, rheological, and microbiological parameters.

### 2.4. Chemical and Instrumental Analyses

The chemical composition and the fatty acid profile of milk (FRS and CTL) were determined; in particular, protein (% *w*/*v*), lactose (% *w*/*v*), and fat (% *w*/*v*) contents were measured by Kjeldahl [20], enzymatic method (Lactose Assay kit, Sigma-Aldrich, Germany), and Gerber [20] methods, respectively. Furthermore, total solids were calculated by drying at 102 °C [21] and solid-not-fat as the difference between total solids and fat content. Chemical and instrumental analyses of semi-finished products (C and T0) and cheeses (MI and MT of ripening) were carried out. The chemical composition and the fatty acid profile were determined on grated cheese taken 2 cm from the rind. Moisture, NaCl, and protein contents were measured by oven drying, Mohr, and Kjeldahl methods, respectively [20]. The extraction of fat and the subsequent GC analysis to determine composition of fatty acid were performed as previously described by Romano et al. [22]. The pH measurements were carried out using a digital pH meter (Crison-Micro TT 2022, Crison Instruments, Barcelona, Spain) in a slurry prepared by macerating 10 g of grated cheese in 10 mL of deionized water. Water activity (a_w_) was measured by Aqualab 4 TE (Decagon Devices Inc., Pullman, WA, USA). 

Instrumental texture was performed on the three points (internal, middle, and external area) of the ray of the slice of semi-product and cheese; average values of points were used for statistical analysis. The compression test was performed by an EZ-Test texturometer Shimadzu (Shimadzuuz Corporation, Kyoto, Japan). The speed and the load cell were 50 mm/min and 500 kg, respectively, and each sample underwent 2 cycles of 50% compression. According to the CIELAB system, the color was determined by using the Konica Minolta CR 300 colorimeter (Minolta, Osaka, Japan). Measurements were carried out on the external (rind) and internal (internal layer) surface of each sample (four measurements for each surface). 

#### 2.4.1. Lipolysis and Oxidative Stability in Cheese

The extent of lipolysis in cheese during ripening was evaluated by measuring the free fatty acids (FFA) concentration, calculating by titrating the acidity of the cheese fat with NaOH 0.1N, according to the method of De Luca et al. [23]; FFA results were expressed as percentage of oleic acid. Lipid oxidation was assessed by measuring the number of peroxide values (PV) according to De Luca et al. [23]; PV was expressed as meqO_2_/kg fat. Thiobarbituric acid reactive substances (TBARs) were measured according to the method adopted by Ambrosio et al. [24]. TBARs were recorded at 532 nm using a Jasco V-530 spectrophotometer (Jasco, Tokyo, Japan) and the results were expressed as mg malondialdehyde/kg sample (ppm).

#### 2.4.2. Fatty Acid Profile

A cold transmethylation was performed on fat in agreement with Romano et al. [22] with some modification. For the preparation of fatty acid methyl esters, approximately 150 mg of lipid were weighed into a conical vial and dissolved in 3 mL of n-hexane and the mixture was shaken vigorously for 30 s. An aliquot (1.5 mL) of the solution was transferred into a vial and 300 µL of methanolic KOH (c = 2 mol/L) were added. The mixture was shaken vigorously for 30 s three times. One (1) µL of the supernatant was analyzed by gas-chromatography (MASTER GC) with FID detector, CP-Select CB capillary column for FAME (length 100 m, internal diameter 0.25 mm, film thickness 0.2 µm) and a temperature-programmed vaporizer (PTV) injector. The injection operating conditions were 50 °C for 0.1 min, 400–260 °C and a hold at 260 °C for 5 min. The detector temperature was set at 260 °C. Helium was used as carrier gas at a flow of 1.2 mL/min. The temperature program of the column was 140 °C for 5 min and subsequent increased at 4 °C/min up to 240 °C where it was held for 18 min. Fatty acid methyl esters were identified by comparing the retention times of the peaks in the sample with previously run pure standard compounds (Supelco^®^ 37 Component FAME Mix, 47885U-Supelco, Sigma-Aldrich, St. Louis, MO, USA). Conjugated linoleic acid (CLA) isomers were identified using an external standard (Sigma-Aldrich, MI, USA, IT). The amount of each FA was expressed as peak area percentage of total fatty acids. The following parameters were calculated: saturated (SFA), monounsaturated (MUFA) and polyunsaturated (PUFA) fatty acids; short-chain fatty acids (SCFA), medium-chain fatty acids (MCFA), and long-chain fatty acids (LCFA); the ratio of omega-6 (n-6) to omega-3 (n-3) fatty acids.

### 2.5. Microbiological Analyses

To enumerate spoilage microorganisms, ten grams/milliliters of each sample were added to 90 mL (1:10 *w*/*v* or *v*/*v*) of sterilized Peptone Water (PW, Oxoid, Madrid, Spain) in a sterile stomacher bag and homogenized for three minutes at 230 rpm using a peristaltic homogenizer (BagMixer^®^400 P, Interscience, Saint Nom, France). Then, ten-fold serial dilutions of each homogenate were prepared in PW, followed by bacterial enumeration for: (a) total aerobic bacterial (TAB 30 °C) counts according to ISO 4833-1:2013 on plate count agar (PCA, Oxoid, Madrid, Spain) incubated at 30 °C for 48/72 h; (b) psychotropic aerobic bacterial counts (TAB 7 °C) on PCA incubated at 7 °C for 10 days [25]; (c) total coliforms according to ISO 4831:2006 on violet red bile lactose agar (VRBL, Oxoid, Madrid, Spain) incubated at 37 °C for 48 h; (d) total Enterobacteriaceae according to ISO 21528-2:2017 on violet red bile glucose agar (VRBG, Oxoid, Madrid, Spain) incubated at 37 °C for 48 h; (e) β-glucuronidase-positive *E. coli* according to ISO 16649-2:2001 selectively isolated on Tryptone Bile X-Glucuronide (TBX, CM0945, Oxoid) incubated at 44 °C for 24/48-h; (f) *Enterococcus faecalis* on KAA (kanamycin aesculin azide, Oxoid, Madrid, Spain) at 37 °C for 48 h; (g) presumptive *Pseudomonas* spp. according to ISO 13720:2010 on Cephalothin-Sodium Fusidate-Cetrimide Agar with Modified CFC Selective Supplement (CFC, CM0559B with SR0103E, Oxoid) incubated aerobically at 25 °C for 48 h; (h) coagulase positive staphylococci according to ISO 6888-1:1999 on Baird-Parker agar (Oxoid, Madrid, Spain) at 37 °C for 24/48 h; (i) lactic acid bacteria (LAB) according to ISO 15214:1998 on De man, Rogosa and Sharpe agar (MRS, CM0361, Oxoid) incubated aerobically at 30°C for 72-h; and (j) yeasts and molds on Dichloran Rose-Bengal Chloramphenicol Agar (DRBC, Oxoid, Madrid, Spain) incubated at 25 °C for 120/168-h, according to ISO 21527:2008. After incubation and counting, the data were expressed as logarithms of the number of colony-forming units (cfu/g) and means and standard error were calculated. 

For the detection of relevant food pathogenic bacteria, 25 g/milliliters of each sample were homogenized into 225 mL (1:10 *w*/*v* or *v*/*v*) of buffer peptone water (BPW, CM0509, Oxoid) and incubated at 37 °C for 24 h for the detection of *Salmonella* spp., in 225 mL Half Fraser broth (HF, CM1053, Oxoid) and incubated at 30 °C for 24 h for the detection of *Listeria monocytogenes*, and in 225 mL Peptone Sorbitol Bile Broth (PSB, 17192, Sigma-Aldrich) for the detection of *Yersinia enterocolitica*. The following steps were carried out in agreement with the mentioned standard methods (ISO 6579-1:2017, ISO 11290-1:2017, and ISO 10273:2017 for *Salmonella* spp., *Listeria monocytogenes*, and *Yersinia enterocolitica*, respectively).

All isolates of presumptive pathogenic bacteria were analyzed by MALDI-TOF MS (MALDI Biotyper^®^ Sirius) first using the “direct colony identification method” [26]. Briefly, colonies were smeared in duplicate onto a 96-pointsteel plate (Bruker Daltonics, Bremen, Germany). Then, samples were covered with a 1 µL matrix solution containing 10 mg/mL of α-cyano-4-hydroxycinnamic acid in acetonitrile (Sigma-Aldrich, Berlin, Germany), deionized water and trifluoracetic acid (50:47.5:2.5, [*v*/*v*/*v*]). Bruker’s Bacterial Test Standard (BTS Bruker Daltonics) was used as reference standard for the mass calibration and Flex Control 3.4 software (Bruker Daltonics, Bremen, Germany) was set in a linear positive ion detection mode (Bruker Daltonics). Isolates were analyzed by matching the collected spectra to those containing in the Bruker MSP database (MBT Compass Library) using the Bruker Compass software at default settings. The identification score criteria were classified according to Jeong et al. [27]: a score ≥ 2.3 indicates highly probable species identification, between 2.0 and 2.3 genus identification and probable species, a score between 1.7 and 1.99 indicates probable gender and <1.7 non-reliable identification.

### 2.6. Statistical Analyses

Statistical analyses were performed using SPSS program, version 27 (IBM Analytics, Armonk, NY, USA). Physicochemical, rheological and color parameters, and microbiological data were statistically analyzed with generalized linear mixed model (GLMMs), including fixed effects of diet (CTL and FRS), and ripening method (MI and MT); furthermore, means were compared using the Tukey test (*p* < 0.05; *p* < 0.01). All experiments were performed at least three times and data were presented as the mean (M) ± standard error (se). 

## 3. Results and Discussions

### 3.1. Chemical and Instrumental Analyses

The first step necessary to evaluate the effect of the ingredients included in the animal diet on the composition of their tissues and/or secretions, such as milk, is the chemical analysis. Indeed, by simply characterizing the chemical composition of the milk used in this study, useful and interesting information was collected. In particular, the compositional analyses of raw milks were carried out to study the matrix in the pre-characterization phase (Table S2). Not less important are the analytical studies for the assessment of the quality of cheeses, which differ from each other in the origins of milk (two experimental buffalo groups) and in the ripening method adopted. As for the milk, the feeding system did not affect its protein content. However, milk fat was found to be significantly higher in L-FRS milk of both batches milk used to produce cheeses (*p* < 0.01). This result could be explained by considering the supplementation of bypass fat (Table S1) only in diet of FRS buffalo group. Indeed, according to Naik [28] the addition of bypass fat does not interfere with rumen fermentation processes and provides more suitable energy to the animal for greater milk synthesis with increased total milk fat. Interestingly, this difference in fat content between CTR and FRS samples was always preserved in cheeses (Table 2), where significant difference in protein content was also detected (*p* < 0.01). Semi-hard cheeses MT were an exception, where the long ripening process (traditional ripened method) eliminated these significant differences. Regarding the moisture, differences existed between CTR and FRS samples, with higher values in the latter group. However, at the end of both ripening processes, these differences were not significant, and the moisture content decreased equally in both groups (CTR and FRS), as a consequence of the natural tendency to de-wheying and the influence of the same environmental drying conditions [29]. As far as ripening methods are concerned, the two ripening techniques have differently influenced the moisture percentage of the two cheese typologies. The long ripening of semi-hard cheese has eliminated any differences in moisture between cheeses ripened with traditional and innovative methods. As reported by Rani and Jagtap [30], this could be due to the balance that is created between adsorption of water by amino groups produced by secondary proteolysis and desorption of water resulting from osmotic pressure. Contrariwise, the moisture content of dry ricotta cheese was considerably (*p* < 0.01) affected by the different ripening methods, showing a lower moisture value in MT cheeses. This phenomenon was in line with a_w_ values, which were lower in MT samples (*p* < 0.01) (results discussed in “Section 3.2” subparagraph). In this regard, the salt diffusion towards the inside of the cheese, the progressive formation of low molecular weight compounds (such as lactic acid), and of non-protein N compounds in more advanced maturing stages could affect the moisture decay in MT dry ricotta cheeses. As was to be expected, the NaCl content showed in all samples upward trends opposed to those of moisture. 

Texture is one of the most important attributes that helps determine the identity of a product, particularly in cheese. Moisture and fat content, salt, pH and degree of proteolysis [31] as such as environmental ripening conditions [32,33] influence the texture of cheeses. The results relating to the determination of the texture are shown in Table 3. Texture profile analysis revealed an increase in hardness, gumminess, and chewiness at the end of the ripening process in all cheeses. Important changes in texture parameters were significantly due to the ripening method (traditional or innovative) adopted. The hardness showed initial low values that increased over the time due to the drying process during ripening [31]. In fact, the observed increase in hardness is related to loss of moisture [33], more evident in dry ricotta cheeses (51.02 ± 1.08 in MI-CTL vs. 18.39 ± 0.29 in MT-CTL; 68.87 ± 0.69 in MI-FRS vs. 20.03 ± 1.20 in MT-FRS; *p* < 0.01). This result is in accordance with Álvarez and Fresno [34] who reported a negative correlation between the moisture content and the hardness of cheeses. Gumminess and chewiness showed similar trends with respect to hardness; these findings were found consistent with the observation of Gebreyowhans et al. [29]. As discussed above for moisture content, no significant differences were found between the MI and MT semi-hard cheeses. Significant effects of diet were observed for texture analysis; indeed, hardness, chewiness, and gumminess and all were found significantly (*p* < 0.01) higher in the FRS cheeses. These results could be explained taking into account the proteins concentration and their degree of hydrolysis in FRS cheeses (Table 2); these conditions influence the macro- and microstructure of cheeses and, consequently, their rheology, increasing the firmness and the fracture stress [35].

Cheese color is an important quality parameter which influences the consumer choice [36] and could be related to the cheese ripening time and conditions. The results of the effect of ripening and feeding systems on the external and internal cheeses color are shown in Table 4. As has frequently been reported in literature, both internal and external lightness (L*) decreased significantly (*p* < 0.01) at the end of ripening [37]; particularly, this trend was more evident on the external surface of cheese [34]. The decrease in lightness in cheeses could be due to an increase in the hydration of proteins and a reduction of light scattering related to free moisture [38]. However, dry ricotta cheeses were an exception, considering that cheeses ripened with the innovative method were characterized by higher lightness values (*p* < 0.01) than both semi-finished products and MT cheeses. This finding could be explained taking into account the similarities in moisture content between semi-finished products and MI cheeses; this aspect could justify the high L* values of the latter cheeses, as moisture is positively correlated to lightness [38]. As regards the semi-hard cheeses, both ripening systems equally affected the samples lightness (*p* < 0.01); indeed, no significant differences were found between MI and MT cheeses, with the exception of CTL-MI and CTL-MT for which a slight (even if significant) difference was detected. In agreement with other authors, as a result of the decrease in lightness there was a slight increase yellowness (b*) values at the end of cheese ripening (Table 4) [34]. An increase in redness (a*) has also been described on the outer face of ripened cheeses. Overall, while significant differences were found, the feeding system did not appear to cause a specific color change. In fact, the inclusion of forage in diet would seem to determinate an increase in b* of semi-hard cheese at T0; however, this result was not repeated for dry ricotta cheese. Therefore, given the non-repeatability of the data, it is possible to hypothesize that the differences described may not be related to the diets.

#### 3.1.1. Lipolysis and Oxidative Stability in Cheese

The FFAs (free fatty acids) determination is a measure of lipolysis and represents the content of free fatty acids dissolved in a certain amount of fat and it is related to the sensory quality of the cheeses. Table 5 shows the evolution of lipolysis in cheese samples. 

Fat hydrolysis showed an increasing trend during the ripening time (higher in MT than in MI) and pointed out an influence of the feeding system on the levels of catabolites. In particular, FFAs showed on average higher values (*p* < 0.01) in FRS cheeses than in CTL; this result could be justified taking into account the high moisture content in FRS cheeses [10] and the present of both indigenous and microbial lipases in FRS raw milk. Indeed, the hydrolyzation of triglycerides, which occurs during cheese ripening by the action of indigenous and bacterial lipases, depends on moisture content, temperature, metal ions contamination and lipase concentration [10]. Free fatty acids that are produced during the primary stages of lipolysis are further degraded into flavoring compounds during the secondary and tertiary stages of cheese ripening [39], positively influencing the flavor of FRS cheeses. Overall, peroxide values of cheeses showed an unstable trend, even if strongly influenced by the feeding system adopted, especially when the traditional ripening method is applied. The lowest peroxide values were observed in FRS cheeses which, on the contrary, showed on average higher TBARs values compared to CTL cheeses. This phenomenon could be explained by the effect of green feed influencing the fatty acid composition of milk by increasing the polyunsaturated fat content [40], which are known to be more susceptible to oxidation. Although lipid oxidation is a quality issue in processed dairy products [41], our results showed low levels of lipid oxidation in the samples studied, especially in those subjected to a shorter innovative ripening method (*p* < 0.01).

#### 3.1.2. Fatty Acid Profile

Ripening methods and feeding systems had a pronounced effect on fatty acid profile and nutritional indices of cheeses. The analysis of fatty acids (FAs) in semi-hard cheeses (Table 6) and dry ricotta cheeses (Table 7) showed that the most abundant FAs were palmitic acid (C16:0), oleic acid (C18:1), myristic acid (C14:0) and stearic acid (C18:0). These findings are in agreement with those of Uzun et al. [42]. Some authors reported that during the long period of ripening, the level of fatty acids increases depending on the ripening time and the type of cheese [43,44]. The short ripening period of dry ricotta cheeses has a low influence on their fatty acids profile which, at the end of ripening, showed few changes compared to the milk FAs profile. However, significant differences have been observed between FRS-MI and FRS-MT dry ricotta cheeses; indeed, short (SCFA) and medium (MCFA) chain fatty acids increased significantly (*p* < 0.01) at the end of the traditional ripening method (MT cheeses). Regarding the semi-hard cheeses, the long ripening period influenced the FAs profile which was found different from that of milk. The most important changes concerned the profile of C18 fatty acids. In agreement with Mureşan et al. [44], oleic acid (C18:1n-9) significantly increased at the end of ripening with values from 20.30% in L-CTL milk to 20.98% in MI-CTL and 22.87% in MT-CTL cheeses (*p* < 0.01) compared to values from 19.53% in L-FRS milk to 25.85% in MI-FRS and 25.11% in MT-FRS cheeses (*p* < 0.01). Oleic acid was followed by stearic acid (C18:0) that significantly increased at the end of ripening with values from 10.44% in L-CTL milk to 11.33% in MI-CTL and 11.13% in MT-CTL cheeses (*p* < 0.05) compared to values from 9.82% in L-FRS milk to 14.76% in MT-FRS and 14.68% in MI-FRS cheeses (*p* < 0.01). Butyric acid (C4:0) showed decreasing values at the end of ripening period (from 2.97% in L-CTL milk to 1.36% in MI-CTL and 1.24% in MT-CTL compared to values from 3.79% in L-FRS milk to 1.65% MI-FRS and 2.41% in MT-FRS; *p* < 0.01) suggesting its selective release by lipases present in cheese or its synthesis by the specific microflora of cheese [45].

It is very important to note that the amount of saturated fatty acids (SFA) in semi-hard cheeses, recognized as health risk factors, significantly decreased with ripening from 73.58% in L-CTL milk to 70.26% in MI-CTR cheeses and 69.20% in MT-CTR cheeses (*p* < 0.05) compared to values from 74.40% in L-FRS milk to 65.50% in MI-FRS cheeses and 67.93% in MT-FRS cheeses (*p* < 0.01). By contrast, the decrease in SFA levels, MUFA and PUFA contents of cheeses increased significantly if compared to those of milk (Tables S3 and S4), highlighting the positive effect of the long ripening [44,46].

Among the PUFA detectable in cheeses, the isomers of CLAs are known for their important biological effects, including anti-carcinogenic and anti-atherogenic properties [47,48]. It is worth noting that the CLAs seemed to show an upward trend during the ripening both in dry ricotta and semi-hard cheeses. These results could be explained by considering the key role of proteins in the production of CLA. Acting as hydrogen donators, they provide protons which react with conjugated structures of radicals (produced by linoleic acid oxidation) to form CLA [49]. Consistent with these results, Buccioni et al. [50] and Govari et al. [46] reported higher values of C18:2 *cis*-9, *trans*-11 CLA in Pecorino Toscano ovine cheese and Kefalotyri cheese than in the respective curds, after the first month of ripening. However, in contrast to these findings, Kumar et al. [51] reported a decrease in the CLAs level during ripening of cheddar buffalo cheeses. In this regard, it is important to underline that many factors, such as the origin and the composition of the milk, the dairy technology, and the applied method of ripening, influence the activities of endogenous enzymes, making it difficult to compare the results of different studies. Overall, no significant differences in FAs have been observed between the semi-hard cheeses ripened with the two methods. Some authors consider changes in the fatty acid profile as a useful verification of the degree of lipolysis [10] and, for this reason, they could play an important role in monitoring the biochemical changes which occur in dairy products [52]. 

Animal nutrition has a greater influence on the fatty acid profile of raw milk and cheeses. According to Salzano et al. [6], feeding with green forage reduced the proportions of C14:0 in fat of milk and cheeses compared to feeding without green ryegrass (Table 6 and Table 7). The lower percentage of C14:0 may be due to inhibition of *de novo* C14:0 synthesis, as a great amount of long chain fatty acids is available in green forage (C16:0 to C18:4) [53]. PUFAs contained in green feed are generally biohydrogenated by rumen bacteria to SFAs and other intermediate products of MUFAs and PUFAs. Since biohydrogenation is incomplete, several digested MUFAs and PUFAs enter the blood plasma and are excreted by the mammary gland in milk [53] increasing unsaturated fatty acids. Buffaloes fed with green forages produce milk rich in MUFAs and PUFAs [54] resulting in healthier productions, characterized by higher nutritional value. The inclusion of green feed influenced the PUFA, MUFA and CLA contents which were higher in FRS cheeses compared to CTL cheeses. 

### 3.2. Microbiological Profile 

Many parameters are usually used to assess the suitability of milk to produce cheese, as the importance of the quality on processing performance is well known. The expansion of the buffalo cheese market to a large panel of products that differ from the popular mozzarella in terms of applied technologies and organoleptic characteristics, requires further and in-depth studies that could clarify the role of two important factors on the microbiological quality of milk, such as animal feeding and milking management. For this reason, one of the objectives of this study was to investigate the potential effect of the diet on the quality of raw matrix which is reflected on that of the end products. Several authors have demonstrated the strong link between the typology of feeding and the profile of the microbial communities of rumen ecology [55,56], however it is not sufficiently clear whether it may have a relevant role on the survival of specific bacteria in milk. This supposition birded by considering that the buffalo milk has been shown to be an important source of bioactive peptides and most of them have been identified and characterized [57,58], revealing disparate activities including antimicrobial activity. For instance, Zhao and colleagues [59] successfully isolated a novel antimicrobial peptide (AMP) from buffalo casein; however, according to the literature, milk AMPs require to be released by enzymatic hydrolysis operate by proteases. For this reason, although the presence of these molecules has been demonstrated in buffalo milk, it is not possible to hypothesize their antimicrobial effect on the microbial communities of fresh milk even if their concentration could be increased by implementing and adjusting the animal diet with green feed. Indeed, as shown in Table 8 and Table 9, microbiological results of raw matrices pointed out an equality and homogeneity of milk samples obtained from both animal groups. In particular, the slight differences in bacterial contamination described for the milk samples used to produce semi-hard cheeses were not consistent with the results of milk used for dry ricotta cheeses. These findings have addressed attention to the analysis of the burden of improper and incorrect management of the milking phase on the microbiological quality of the milk. The milking is a known critical point in the production chain due to sensitivity and dependence of the practice quality on the application of good hygiene procedures. The collected raw milk samples showed very high concentrations of total aerobic bacteria 30° C, total Coliforms and Enterobacteriaceae, above the legislative and quality standard threshold values. In particular, the Regulation (EC) No 853/2004 of the European Parliament and of the Council set the limit of TAB 30 °C for raw milk from species other than cow at 1 500 000 cfu/mL. These microorganisms represent important indicators because their concentrations reflect the degree and severity of the microbiological contaminations along the production process. The TAB 30 °C counts for all milk samples under study appear to suggest an important and serious flaw that inevitably affected the milk phase management. Our data exceeded both legislative limit and literature data [60,61] reaching values between 7.68 ± 0.37 and 10.71 ± 0.17 Log (cfu/mL) for the milk used to produce dry ricotta and semi-hard cheeses, respectively. Based on these results, it is possible to affirm that milk samples were probably affected by environmental bacterial contamination. To clarify and justify this hypothesis, the total Coliforms and Enterobacteriaceae values were taken into consideration. In fact, a close interconnection among the aforementioned bacterial communities has been described, recording such a high concentration of the so-called hygiene indicators bacteria that necessarily affected the levels of total mesophilic bacteria (TAB 30 °C). The contamination of milk could be attributed to several factors which could be summarized in improper staff training. The personal hygiene of milk workers as well as the disinfection of equipment and milking area and the application of adequate and standardized procedures depend on the skills and competences of the people actively working in this critical phase. The enormous presence of *Escherichia coli* (about 5 Log (cfu/mL)) in the milk used for the manufacture of dry ricotta cheeses is a clear sign of inadequate hygienic conditions. In conclusion, as far as milk concerns, despite the dramatic scene, none of the investigated samples were found to be positive for *Salmonella* spp., *Listeria monocytogenes* and *Yersinia enterocolitica*. 

As regards the microbiological analyses carried out both on semi-finished products of semi-hard cheeses and end-products (dry ricotta cheeses and semi-hard cheeses), the purpose of this study was threefold, having the specific intent of (i) investigating the quality of the supply chain, (ii) the differences in microbiological communities between cheeses in consideration of the origin of milk, coming from the two experimental groups of animal (different diets), and (iii) the different ripening technologies adopted. Of course, the interpretation of data took into account all of these factors and some results were closely related to more than one of them. In agreement with the collected results on milk samples, a green feed diet does not seem to make a selection on the families and genera of microorganisms sought in cheeses, recording no significant differences between cheeses produced with milk FRS and CTR. In this regard, it is considered appropriate not to exclude the possibility that the integration of green feed into the animal diet may affect the composition of the bacterial community (i.e., the abundance of specific species of lactic acid bacteria) and a further and in-depth metataxonomic analysis is necessary to clarify this aspect. As far as ripening technology concerns, very interesting results have been obtained. Aware of the own chemical properties of buffalo milk which affect the yield of cheese and the duration of the ripening phase [10], the main challenge of this study was to demonstrate that the novel method could reduce the ripening period of buffalo cheeses (and, therefore, the production costs) without diminishing food safety and hygiene, and organoleptic benefits guaranteed by traditional technologies. It is well known that cheese ripening is an outcome of processes responsible for the lysis of glucose, proteins, and lipids in the matrix, allowing to obtain the desirable rheological properties. To this regard, an important scientific contribution to better understand the biochemical and metabolic reactions occurring in the matrix during the ripening was provided by Watkinson et al. [11] who clarify the key role of cheese pH on all the rheological properties, especially on the texture. Furthermore, the known ability of pH to influence the vitality and growth of microbiological communities is considered, it is easy to understand the choice of the authors to test in this study a new ripening technology based on the continuous monitoring of the pH of the cheese. 

Milk and dairy products are often linked to cases of foodborne disease [9], many of which related to the presence of dangerous pathogenic bacteria, such as *Listeria monocytogenes*, *Salmonella* spp., and *Escherichia coli* (Shiga toxin-producing, STEC). The risk associated with the consumption of contaminated cheeses is expressed above all for unpasteurized and soft cheeses, both for the cheese-making and for the ripening methods that do not allow for the slow down and control of the concentration of specific bacteria. In wanting to analyze the consequences of a short ripening, it is necessary to consider how and to what extent the water activity and the moisture are affected by reducing the maturation period. These two intrinsic factors in food weigh on the survival of pathogenic bacteria and by manipulating and controlling their levels it is possible to transform foods into bad growing substrates. According to the literature [62] the presence of foodborne pathogens has been documented mainly in cheeses with a percentage of moisture ≥ 50% (soft-cheeses) and less frequently in semi-soft and hard cheeses (>39%–<50% and ≤ 50% of moisture content, respectively). One of the oldest and most common tools capable to reduce the levels of a_w_ and moisture is the long ripening. Indeed, as could be expected, in this study the values of a_w_ as well as moisture were found to be lower in cheeses ripened according to the traditional methods (Table 2, Table 8 and Table 9), especially for dry ricotta cheeses. For the semi-hard cheeses, the decrease of these parameters occurred with the same intensity in the cheeses cured with the two methods (Table 2, Table 8 and Table 9), affecting the survival of the bacterial cells of *Salmonella* spp. both in MI and MT samples, even if isolated in the curd and in the semi-finished products (T0) and identified with MALDI TOF. These findings are consistent with those published by several authors [63] who have demonstrated that specific spoilage and pro-technological bacteria also play a crucial role in inhibition of the growth of pathogenic bacteria. Indeed, their absence in matured cheeses could be due to the production of various metabolites with antimicrobial activities (e.g., bacteriocins, organic acids and peroxide) by the microorganisms that inhabit the matrix, such as Enterococcus faecium and *E. faecalis* [64], lactic acid bacteria (*Lactobacillus*, *Leuconostoc* and *Weissella*) [65], and yeast [66]. Therefore, according to the microbiological results of semi-hard buffalo cheeses, it is possible to hypothesize that the increase in concentration during the period of maturation of both *Enterococcus* and Lactic acid bacteria may have contributed to inhibiting the survival of *Salmonella* spp. in ripened cheeses. Furthermore, it is worth noting that the absence of *Salmonella* spp. in milk, but only in curds and T0 samples, suggests an environmental contamination which occurs probably during the cheesemaking. Furthermore, despite the bactericidal effect obtained with both ripening technologies, the presence of these pathogenic bacteria in the supply chain is to be considered alarming for food business operators who have an obligation to protect the health of consumers. *Listeria monocytogenes* and *Yersinia enterocolitica* were not detected in any of the sample.

Once it was demonstrated that the innovative method, although characterized by a shorter ripening period, does not lose the ability to hinder the growth of pathogenic bacteria, the next and last step was to investigate how the differences in climatic recipes could influence the microbiologic profiles of both cheeses under study. Considering the differences in activity water levels which exist between dry ricotta cheeses ripened with the two methods, it is understandable whether significant differences in microbial counts were found. However, as shown in Table 9, no significant differences were detected between cheeses belonging to the two experimental groups (MI and MT), with the exception of a slight difference in total mesophilic bacterial count (TAB 30 °C), lactic acid bacteria and in *Enterococcus* concentrations (Table 9). As anticipated, this event is completely normal if the four-fold differences in duration of the two ripening technologies are taken into account; nevertheless, it is interesting to note the compromise reached with the novel method which guarantees a microbiological profile not far from that obtained with the long ripening. By consulting the results collected for those factors that hinder the microbial growth (i.e., a_w_, NaCl content, moisture, pH), it is possible to hypothesize that both the decrease of the pH and the relative increase of salt percentage may have contributed to controlling microbiological replication. However, it is not possible to ignore the presence of coagulase-positive staphylococci (*Staphylococcus aureus* and other species) in the samples already on the first day of production; their presence in dry ricotta as well as in semi-hard cheeses suggests the application of incorrect handling procedures during the manufactures, as they recognized as hygiene markers (Reg. CE 2073/2005). Although current legislation has not set legislative limits for *Staphylococcus aureus*, the isolation and identification (MALDI TOF) of several colonies of this bacterium from semi-hard cheeses is alarming. The weight of this microorganism is well-known and documented among those responsible for food borne disease, and some of the clear consequences of the ingestion of toxin produced by *S. aureus* are collected and summarized into the annual reports of EFSA and ECDC as numbers of human cases, hospitalizations, and outbreaks per year [9]. In conclusion, as far as semi-hard cheese concerns, the effect of the innovative ripening method to hamper the growth of spoilage bacteria could be superimposed on that obtained with the traditional protocol (Table 8). The intrinsic factors, which commonly collaborate in the creation of the most effective and natural obstacle system towards the growth of microorganisms, have been equally modified. Subsequentially, by adjusting the climatic recipe, it was possible to achieve the same microbiological results even reducing the ripening period by two-thirds. Furthermore, looking at the collected results in detail, it seems that, paradoxically, better results in the containment and reduction in the concentration of bacteria usually referred as hygienic indicator (i.e., Enterobacteriaceae, total coliforms, and *E. coli*) have been obtained.

## 4. Conclusions

In view of the economic and nutritional value of buffalo dairy products, novel techniques should be investigated to improve the sustainability of buffalo cheese manufacture. The results of this study showed that the choice of suitable climatic ripening conditions based on the continuous control and monitoring of all parameters, allows to considerably reduce the duration of the technological process, especially in the case of semi-hard and hard cheeses (i.e., semi-hard buffalo cheese). This goal was achieved without negatively compromising any of the desirable physicochemical properties and the safety and hygiene of the final products. Indeed, although the ripening time has been reduced, the “fast” method contributed to hinder the growth of unwanted bacteria. Moreover, the inclusion of green feed in buffalo diet significantly affected the chemical composition and the fatty acids profile (high content of MUFAs and PUFAs) of both raw and transformed products, enhancing their healthier and nutritional value. Therefore, accelerated ripening methods and functional diets could be candidates as promising systems for the valorization of buffalo cheeses in the dairy industry; the protocols developed will help ensure more sustainable production and the expansion of the buffalo milk processing sector. 

## 5. Patents

Stagionello—European Patented Device and controlled pH—n. EP 2769276 B1.

## Figures and Tables

**Table 1:** Experimental ripening design for dry ricotta cheese and semi-hard cheese.

Samples	Ripening Time	Ripening Steps	pH	Air Temperature (°C)	RH (%)	Airflow (m/s)	References
Dry ricotta cheese	INNOVA—TIVE 7 days	Dripping (2 h)	5.5	+35	60	1	This study
		Stewing (20 h)	5.5	+22	40	1	
		Drying 1 (24 h)	5.0	+20	45	1	
		Drying 2 (24 h)	5.0	+20	42	1	
		Drying 3 (24 h)	5.0	+18	41	1	
		Drying 4 (24 h)	5.2	+16	40	1	
		Drying 5 (24 h)	5.3	+14	38	1	
		Drying 6 (24 h)	5.4	+12	36	1	
		Ripening (24 h)	5.5	+8/10	35	1	
	TRADITIONAL 30 days		-	+4		-	[18]
Semi-hard cheese	INNOVATIVE 43 days	Drying 1 (24 h)	4.8	+24	75	0	
		Drying 2 (24 h)	5.0	+23	76	0	This study
		Drying 3 (24 h)	5.0	+21	78	0	
		Drying 4 (24 h)	5.1	+19	80	0	
		Drying 5 (24 h)	5.2	+17	82	0	
		Drying 6 (24 h)	5.3	+15	83	0	
		Drying 7 (24 h)	5.4	+13	84	0	
		Drying 8 (24 h)	5.5	+12	85	0	
		Ripening (35 days)	5.6	+11	75	0	
	TRADITIONAL 90 days		-	+14/15	90–96	-	[19]

**Table 2:** Effects of feeding system and ripening time on chemical composition of dry ricotta cheeses and semi-hard cheeses.

	Semi-Hard Cheese					Dry Ricotta Cheese		
		Semi-Finished Products		Ripened Cheeses		Semi-Finished Product	Ripened Cheeses	
Item		C	T0	MI	MT	T0	MI	MT
Fat, %	CTL	23.58 ± 0.10 ^X,A^	32.28 ± 0.16 ^X,aB^	43.73 ± 0.51 ^X,aC^	38.25 ± 2.26 ^bB^	15.14 ± 0.51 ^X,A^	27.92 ± 1.16 ^X,B^	40.04 ± 1.43 ^X,C^
	FRS	24.03 ± 0.04 ^Y,A^	35.01 ± 0.39 ^Y,B^	50.26 ± 0.58 ^Y,C^	42.81 ± 0.23 ^D^	12.54 ± 0.39 ^Y,A^	22.99 ± 0.70 ^Y,B^	45.03 ± 0.68 ^Y,C^
Protein, %	CTL	15.18 ± 0.59 ^X,A^	21.98 ± 0.26 ^X,B^	20.07 ± 0.10 ^X,C^	27.50 ± 0.27 ^D^	7.03 ± 0.24 ^X,A^	13.06 ± 0.03 ^X,B^	21.08 ± 0.11 ^X,C^
	FRS	17.89 ± 0.24 ^Y,A^	24.94 ± 0.16 ^Y,aB^	25.80 ± 0.36 ^Y,bB^	26.14 ± 0.63 ^B^	12.54 ± 0.39 ^Y,A^	13.89 ± 0.18 ^Y,B^	22.32 ± 0.17 ^Y,C^
Moisture, %	CTL	52.11 ± 0.34 ^A^	34.77 ± 0.31 ^X,B^	18.47 ± 0.47 ^C^	19.54 ± 0.49 ^C^	67.17 ± 1.70 ^A^	45.19 ± 0.17 ^X,B^	18.39 ± 0.29 ^C^
	FRS	52.72 ± 0.45 ^A^	39.29 ± 0.29 ^Y,B^	18.63 ± 0.14 ^C^	19.61 ± 0.26 ^D^	70.24 ± 1.19 ^A^	49.65 ± 0.57 ^Y,B^	20.03 ± 1.20 ^C^
NaCl, %	CTL	0.25 ± 0.05 ^A^	0.59 ± 0.02 ^X,B^	0.99 ± 0.10 ^C^	1.14 ± 0.04 ^C^	1.76 ± 0.05 ^X,A^	3.5 ± 0.16 ^x,aB^	3.96 ± 0.05 ^X,bB^
	FRS	0.20 ± 0.01 ^A^	0.43 ± 0.05 ^Y,B^	1.03 ± 0.11 ^C^	0.98 ± 0.14 ^C^	2.51 ± 0.17 ^Y,A^	2.88 ± 0.19 ^y,A^	5.58 ± 0.40 ^Y,B^

FRS, group of buffaloes fed with green forage; CTL, group of buffaloes fed without green forage. C, curd; T0, 1 d; MI, time of innovative method; MT, time of traditional method. On each storage day, three samples by experimental group were analyzed. Statistical analysis was performed comparing experimental groups at each sampling time and within each experimental group along the ripening period. All data were presented as mean (m) ± standard error (se). Different superscript uppercase letters indicate a significant difference at *p* < 0.01. Different superscript lowercase letters indicate a significant difference at *p* < 0.05. ^A–D, a–b^ Mean values in the same row (same batch in different weeks) with different letters presented significant differences. ^X–Y^ Mean values in the same column (different samples on the same time/ripening time) with different letters presented significant differences.

**Table 3:** Effects of feeding system and ripening time on texture analysis of dry ricotta cheeses and semihard cheeses.

	Semi-Hard Cheese					Dry Ricotta Cheese		
		Semi-Finished Products		Ripened Cheeses		Semi-Finished Product	Ripened Cheeses	
Item		C	T0	MI	MT	T0	MI	MT
Hardness, Kg	CTL	6.02 ± 0.45 ^X,A^	10.8 ± 0.39 ^X,B^	31.38 ± 1.68 ^X,C^	33.17 ± 0.88 ^X,C^	1.94 ± 0.11 ^X,A^	8.68 ± 0.28 ^X,B^	94.90 ± 1.27 ^X,C^
	FRS	18.75 ± 0.22 ^Y,A^	13.18 ± 0.28 ^Y,B^	45.13 ± 1.68 ^Y,C^	42.71 ± 3.78 ^Y,C^	2.58 ± 0.11 ^Y,A^	11.4 ± 0.27 ^Y,B^	77.94 ± 1.24 ^Y,C^
Springiness, mm	CTL	1.59 ± 0.02 ^X,A^	1.01 ± 0.03 ^X,C^	0.96 ± 0.00 ^B^	0.94 ± 0.00 ^C^	0.83 ± 0.05 ^A^	0.91 ± 0.00 ^A^	4.17 ± 0.85 ^x,B^
	FRS	1.89 ± 0.14 ^Y,A^	1.68 ± 0.08 ^Y,B^	1.08 ± 0.11 ^A^	1.08 ± 0.15 ^A^	0.90 ± 0.04 ^A^	0.89 ± 0.04 ^A^	1.65 ± 0.03 ^y,B^
Chewiness, mm/kg	CTL	5.21 ± 0.26 ^X,A^	6.04 ± 0.13 ^X,B^	13.59 ± 0.31 ^C^	20.08 ± 1.62 ^D^	0.59 ± 0.10 ^X,A^	2.19 ± 0.08 ^B^	19.16 ± 9.42
	FRS	7.67 ± 0.14 ^Y,B^	8.98 ± 0.42 ^Y,B^	18.34 ± 0.89 ^C^	20.72 ± 1.65 ^C^	0.90 ± 0.01 ^Y,A^	2.76 ± 0.14 ^B^	1.94 ± 0.66
Gumminess, N	CTL	3.27 ± 0.12 ^X,A^	4.88 ± 0.18 ^Y,B^	12.40 ± 0.59 ^C^	12.62 ± 0.42 ^C^	0.70 ± 0.10 ^A^	2.58 ± 0.19 ^X,B^	7.87 ± 1.66 ^X,C^
	FRS	7.21 ± 0.58 ^Y,aA^	5.68 ± 0.05 ^Y,bB^	12.36 ± 0.54 ^B^	13.76 ± 0.76 ^B^	0.93 ± 0.03 ^A^	3.57 ± 0.22 ^Y,B^	28.21 ± 1.75 ^Y,C^

FRS, group of buffaloes fed with green forage; CTL, group of buffaloes fed without green forage. C, curd; T0, 1 d; MI, time of innovative method; MT, time of traditional method. On each storage day, three samples by experimental group were analyzed. Statistical analysis was performed comparing experimental groups at each sampling time and within each experimental group along the ripening period. All data were presented as mean (m) ± standard error (se). Different superscript uppercase letters indicate a significant difference at *p* < 0.01. Different superscript lowercase letters indicate a significant difference at *p* < 0.05. ^A–C, a–b^ Mean values in the same row (same batch in different weeks) with different letters presented significant differences. ^X–Y, x–y^ Mean values in the same column (different samples on the same time/ripening time) with different letters presented significant differences.

**Table 4:** Effects of feeding system and ripening time on internal and external color of semi-hard cheeses.

			Semi-Hard Cheese				Dry Ricotta Cheese		
			Semi-Finished Products		Ripened Cheeses		Semi-Finished Product	Ripened Cheeses	
Item			C	T0	MI	MT	T0	MI	MT
External	L*	CTL	87.82 ± 0.24 ^A^	84.94 ± 0.17 ^B^	67.67 ± 0.27 ^X,C^	65.93 ± 0.38 ^D^	89.85 ± 0.50 ^A^	92.35 ± 0.51 ^B^	66.70 ± 2.09 ^C^
		FRS	85.50 ± 1.19 ^A^	85.24 ± 0.18 ^A^	65.54 ± 0.37 ^Y,B^	64.77 ± 0.41 ^B^	89.72 ± 1.14 ^A^	92.88 ± 0.96 ^A^	69.23 ± 0.51 ^B^
	a*	CTR	−1.11 ± 0.10 ^A^	−1.05 ± 0.09 ^X,A^	−0.64 ± 0.42	0.31 ± 0.26 ^X,B^	−1.51 ± 0.14 ^A^	−0.34 ± 0.17 ^B^	−0.60 ± 0.21 ^X,B^
		FRS	−0.84 ± 0.11 ^A^	−1.74 ± 0.11 ^Y,B^	−1.42 ± 0.29	−1.49 ± 0.13 ^Y,B^	−0.42 ± 0.95	0.16 ± 0.49	0.40 ± 0.21 ^Y^
	b*	CTL	10.89 ± 0.57 ^A^	11.24 ± 0.18 ^X,A^	16.38 ± 0.09 ^X,aB^	18.36 ± 0.72 ^X,bB^	8.98 ± 0.22 ^A^	23.20 ± 0.97 ^B^	23.51 ± 0.52 ^X,B^
		FRS	11.54 ± 0.72 ^aA^	13.42 ± 0.47 ^Y,bA^	14.07 ± 0.50 ^Y,b^	15.14 ± 0.06 ^Y,B^	8.08 ± 0.39 ^A^	22.77 ± 2.97 ^B^	20.24 ± 0.69 ^Y,B^
Internal	L*	CTL	90.36 ± 0.23 ^A^	87.61 ± 0.50 ^B^	83.49 ± 0.65 ^X,C^	83.78 ± 0.07 ^X,C^	91.35 ± 0.37 ^A^	102.20 ± 0.40 ^X,B^	81.05 ± 0.76 ^C^
		FRS	89.40 ± 0.96 ^aB^	86.76 ± 0.39 ^b^	86.25 ± 0.29 ^Y,B^	86.39 ± 0.32 ^Y,B^	91.33 ± 0.44 ^A^	100.18 ± 0.43 ^Y,B^	79.66 ± 0.46 ^C^
	a*	CTL	−1.04 ± 0.04 ^x,A^	−1.10 ± 0.08 ^X,A^	−1.40 ± 0.24	−1.40 ± 0.07 ^B^	−1.39 ± 0.06 ^A^	−1.36 ± 0.08 ^A^	−3.28 ± 0.03 ^X,B^
		FRS	−1.14 ± 0.02 ^y,A^	−1.55 ± 0.06 ^Y,B^	−1.13 ± 0.04 ^aA^	−1.28 ± 0.08 ^b^	−1.44 ± 0.09 ^aA^	−1.20 ± 0.02 b^A^	−2.30 ± 0.06 ^Y,B^
	b*	CTL	11.27 ± 0.54 ^X,aA^	13.66 ± 0.71 ^X,bA^	20.00 ± 0.63 ^x,B^	19.46 ± 0.24 ^X,B^	8.51 ± 0.02 ^X,A^	11.22 ± 0.26 ^X,B^	15.92 ± 0.13 ^X,C^
		FRS	13.03 ± 0.27 ^Y,A^	16.37 ± 0.23 ^Y,B^	18.31 ± 0.17 ^y,C^	17.67 ± 0.36 ^Y,C^	7.82 ± 0.10 ^Y,A^	10.27 ± 0.12 ^Y,B^	12.52 ± 0.20 ^Y,C^

FRS, group of buffaloes fed with green forage; CTL, group of buffaloes fed without green forage. C, curd; T0, 1 d; MI, time of innovative method; MT, time of traditional method. L*, lightness; a*, redness; b*, yellowness. On each storage day, three samples by experimental group were analyzed. Statistical analysis was performed comparing experimental groups at each sampling time and within each experimental group along the ripening period. All data were presented as mean (m) ± standard error (se). Different superscript uppercase letters indicate a significant difference at *p* < 0.01. Different superscript lowercase letters indicate a significant difference at *p* < 0.05. ^A–D, a–b^ Mean values in the same row (same batch in different weeks) with different letters presented significant differences. ^X–Y, x–y^ Mean values in the same column (different samples on the same time/ripening time) with different letters presented significant differences.

**Table 5:** Effects of feeding system and ripening time on oxidation index of dry ricotta cheese and semi-hard cheeses.

	Semi-Hard Cheese					Dry Ricotta Cheese		
		Semi-Finished Products		Ripened Cheeses		Semi-Finished Product	Ripened Cheeses	
Item		C	T0	MI	MT	T0	MI	MT
PV, meqO_2_/kg	CTL	1.54 ± 0.01 ^X,A^	1.55 ± 0.01 ^X,A^	1.39 ± 0.00 ^X,B^	1.26 ± 0.01 ^X,C^	1.82 ± 0.02 ^X^	1.72 ± 0.05 ^X^	1.78 ± 0.01 ^X^
	FRS	1.33 ± 0.01 ^Y,A^	1.42 ± 0.01 ^Y,B^	1.74 ± 0.02 ^Y,C^	1.43 ± 0.01 ^Y,B^	1.53 ± 0.03 ^Y,A^	1.28 ± 0.01 ^Y,B^	2.58 ± 0.01 ^Y,C^
FFA, %	CTL	0.08 ± 0.00 ^x,aA^	0.11 ± 0.02 ^bA^	0.63 ± 0.01 ^X,B^	1.08 ± 0.01 ^X,C^	0.04 ± 0.00 ^X,A^	0.09 ± 0.01 ^X,B^	0.10 ± 0.00 ^X,C^
	FRS	0.15 ± 0.03 ^y,A^	0.11 ± 0.00 ^A^	0.69 ± 0.00 ^Y,B^	0.95 ± 0.01 ^Y,C^	0.12 ± 0.01 ^Y,A^	0.12 ± 0.00 ^Y,A^	0.16 ± 0.00 ^Y,B^
TBARs, mg/kg	CTL	0.05 ± 0.00 ^X,A^	0.08 ± 0.00 ^X,B^	0.06 ± 0.00 ^X,A^	0.54 ± 0.04 ^x,C^	0.11 ± 0.01 ^X^	0.05 ± 0.00 ^X^	0.08 ± 0.04 ^X^
	FRS	0.09 ± 0.00 ^Y,A^	0.13 ± 0.01 ^Y,B^	0.13 ± 0.00 ^Y,C^	0.43 ± 0.03 ^y,D^	0.07 ± 0.01 ^Y,A^	0.04 ± 0.00 ^Y,B^	0.02 ± 0.00 ^Y,C^

FRS, group of buffaloes fed with green forage; CTL, group of buffaloes fed without green forage. C, curd; T0, 1 d; MI, time of innovative method; MT, time of traditional method. PV, peroxide value; FFA, free fatty acid; TBARs, thiobarbituric acid reactive substance. On each storage day, three samples by experimental group were analyzed. Statistical analysis was performed comparing experimental groups at each sampling time and within each experimental group along the ripening period. All data were presented as mean (m) ± standard error (se). Different superscript uppercase letters indicate a significant difference at *p* < 0.01. Different superscript lowercase letters indicate a significant difference at *p* < 0.05. ^A–D, a–b^ Mean values in the same row (same batch in different weeks) with different letters presented significant differences. ^X–Y, x–y^ Mean values in the same column (different samples on the same time/ripening time) with different letters presented significant differences.

**Table 6:** Effects of feeding system and ripening time on fatty acid profile of semi-hard cheeses.

		Raw Material	Semi-Finished Products		Ripened Cheeses	
FA, g/100 g of FA		L	C	T0	MI	MT
C4:0	CTL	2.97 ± 0.06 ^X,A^	2.93 ± 0.10 ^x,A^	2.21 ± 0.05 ^X,B^	1.36 ± 0.02 ^X,C^	1.24 ± 0.02 ^X,D^
	FRS	3.79 ± 0.07 ^Y,A^	2.67 ± 0.06 ^y,B^	2.95 ± 0.06 ^Y,C^	1.65 ± 0.02 ^Y,D^	2.41 ± 0.04 ^Y,E^
C6:0	CTL	2.01 ± 0.03 ^A^	1.96 ± 0.06 ^X,A^	1.72 ± 0.04 ^x,B^	1.36 ± 0.02 ^C^	1.24 ± 0.02 ^X,D^
	FRS	2.04 ± 0.05 ^A^	1.70 ± 0.04 ^Y,aD^	1.85 ± 0.04 ^y,bB^	1.39 ± 0.02 ^C^	1.65 ± 0.03 ^Y,D^
C8:0	CTL	0.86 ± 0.02 ^X,A^	1.10 ± 0.04 ^X,B^	0.98 ± 0.02 ^C^	0.87 ± 0.01 ^X,A^	0.87 ± 0.02 ^X,A^
	FRS	1.06 ± 0.03 ^Y,aA^	0.97 ± 0.02 ^Y,bA^	0.99 ± 0.02 ^A^	0.76 ± 0.01 ^Y,B^	1.02 ± 0.02 ^Y,B^
C10:0	CTL	1.88 ± 0.03 ^X,A^	2.20 ± 0.07 ^X,B^	2.08 ± 0.05 ^X,B^	1.86 ± 0.03 ^X,A^	1.73 ± 0.03 ^X,C^
	FRS	2.05 ± 0.05 ^Y,A^	1.82 ± 0.04 ^Y,B^	1.85 ± 0.04 ^Y,B^	1.14 ± 0.02 ^Y,C^	1.27 ± 0.02 ^Y,D^
C12:0	CTL	2.97 ± 0.07 ^X,A^	2.94 ± 0.10 ^X,A^	2.82 ± 0.07 ^X,aA^	2.60 ± 0.04 ^X,Bb^	2.47 ± 0.05 ^X,Ab^
	FRS	2.60 ± 0.05 ^Y,aA^	2.43 ± 0.06 ^Y,aB^	2.46 ± 0.06 ^Y,A^	1.52 ± 0.03 ^Y,C^	1.52 ± 0.30 ^Y,C^
C14:0	CTL	14.65 ± 0.25 ^X,a^	14.83 ± 0.49 ^X^	14.45 ± 0.33 ^X^	13.96 ± 0.23 ^X^	13.79 ± 0.26 ^X,b^
	FRS	13.26 ± 0.25 ^Y,A^	12.91 ± 0.31 ^Y,A^	13.09 ± 0.30 ^Y,A^	8.67 ± 0.14 ^Y,B^	8.68 ± 0.15 ^Y,B^
C14:1	CTL	1.13 ± 0.04 ^X,A^	1.23 ± 0.04 ^X,A^	1.23 ± 0.03 ^X,A^	1.09 ± 0.02 ^X,A^	0.93 ± 0.02 ^X,B^
	FRS	0.97 ± 0.02 ^Y,aA^	0.91 ± 0.02 ^Y,bA^	0.92 ± 0.02 ^Y,A^	0.63 ± 0.01 ^Y,B^	0.32 ± 0.01 ^Y,C^
C15:0	CTL	1.39 ± 0.04 ^X^	1.39 ± 0.05 ^X^	1.39 ± 0.03 ^X^	1.40 ± 0.02 ^X^	1.40 ± 0.03 ^X^
	FRS	1.12 ± 0.02 ^Y^	1.16 ± 0.03 ^Y,A^	1.18 ± 0.03 ^Y,A^	0.88 ± 0.02 ^Y,B^	0.88 ± 0.02 ^Y,B^
C16:0	CTL	34.75 ± 0.72 ^X^	34.01 ± 1.12	34.03 ± 0.78	34.21 ± 0.57	34.32 ± 0.65
	FRS	37.59 ± 0.69 ^Y,aA^	36.61 ± 0.87 ^A^	36.24 ± 0.83 ^A^	33.25 ± 0.55 ^Ab^	35.13 ± 0.6 ^b^
C16:1	CTL	2.68 ± 0.08 ^x,aA^	3.01 ± 0.10 ^X,b^	3.01 ± 0.07 ^X,b^	2.93 ± 0.05 ^X,B^	2.81 ± 0.05 ^X^
	FRS	2.44 ± 0.05 ^y^	2.54 ± 0.06 ^Y^	2.46 ± 0.06 ^Y^	2.19 ± 0.04 ^Y^	2.08 ± 0.04 ^Y^
C17:0	CTL	0.57 ± 0.02 ^A^	0.57 ± 0.02 ^A^	0.57 ± 0.01 ^A^	0.67 ± 0.01 ^X,B^	0.57 ± 0.01 ^A^
	FRS	0.59 ± 0.02	0.56 ± 0.01	0.57 ± 0.01	0.59 ± 0.01 ^Y^	0.59 ± 0.01
C18:0	CTL	10.44 ± 0.19 ^x,aA^	10.33 ± 0.34 ^ac^	10.68 ± 0.25 ^aC^	11.33 ± 0.19 ^X,bB^	11.13 ± 0.21 ^X,bc^
	FRS	9.82 ± 0.19 ^y,A^	11.01 ± 0.26 ^B^	10.81 ± 0.25 ^B^	14.76 ± 0.24 ^Y,C^	14.68 ± 0.26 ^Y,C^
C18:1*n*-9 *trans*	CTL	1.73 ± 0.14 ^A^	0.91 ± 0.03 ^X,aB^	0.99 ± 0.23 ^X,bB^	1.29 ± 0.02 ^X,C^	0.64 ± 0.01 ^X,D^
	FRS	1.62 ± 0.03 ^A^	1.17 ± 0.03 ^Y,B^	1.19 ± 0.03 ^Y,B^	1.60 ± 0.03 ^Y,A^	0.94 ± 0.02 ^Y,C^
C18:1*n*-9 *cis*	CTL	20.30 ± 0.35 ^A^	18.99 ± 0.62 ^X,aB^	19.88 ± 0.46 ^X,bB^	20.98 ± 0.35 ^X,C^	22.87 ± 0.43 ^X,D^
	FRS	19.53 ± 0.44 ^A^	20.12 ± 0.48 ^Y,A^	20.22 ± 0.46 ^Y,A^	25.80 ± 0.43 ^Y,B^	25.11 ± 0.44 ^Y,B^
C18:2*n*-6 *cis*	CTL	1.52 ± 0.04 ^X,a^	1.60 ± 0.05 ^X^	1.60 ± 0.04 ^X^	1.62 ± 0.03 ^b^	1.52 ± 0.03 ^X,ac^
	FRS	1.37 ± 0.03 ^Y,A^	1.31 ± 0.03 ^Y,A^	1.14 ± 0.03 ^Y,B^	1.66 ± 0.03 ^aC^	1.76 ± 0.03 ^Y,bC^
C18:3*n*-3	CTL	0.31 ± 0.01 ^X^	0.31 ± 0.01	0.31 ± 0.01	0.31 ± 0.01 ^X^	0.31 ± 0.01 ^X^
	FRS	0.42 ± 0.02 ^Y,A^	0.30 ± 0.01 ^B^	0.31 ± 0.01 ^B^	0.42 ± 0.01 ^Y,A^	0.53 ± 0.01 ^Y,C^
CLA *cis*-9, *trans*-11	CTL	0.58 ± 0.02 ^X^	0.58 ± 0.02	0.58 ± 0.01	0.59 ± 0.01 ^X^	0.58 ± 0.01 ^X^
	FRS	0.47 ± 0.02 ^Y,A^	0.57 ± 0.01 ^B^	0.58 ± 0.01 ^B^	0.72 ± 0.01 ^Y,C^	0.72 ± 0.01 ^Y,C^

FRS, group of buffaloes fed with green forage; CTL, group of buffaloes fed without green forage. L, raw buffalo milk; C, curd; T0, 1 d; MI, time of innovative method; MT, time of traditional method. FA, fatty acids. On each storage day, three samples by experimental group were analyzed. Statistical analysis was performed comparing experimental groups at each sampling time and within each experimental group along the ripening period. All data were presented as mean (m) ± standard error (se). Different superscript uppercase letters indicate a significant difference at *p* < 0.01. Different superscript lowercase letters indicate a significant difference at *p* < 0.05. ^a–c, A–E^ Mean values in the same row (same batch in different weeks) with different letters presented significant differences. ^X–Y, x–y^ Mean values in the same column (different samples on the same time/ripening time) with different letters presented significant differences.

**Table 7:** Effects of feeding system and ripening time on fatty acid profile of dry ricotta cheeses.

		Raw Material	Semi-Finished Product	Ripened Cheeses	
FA, g/100 g of FA		L	T0	MI	MT
C4:0	CTL	3.92 ± 0.07 ^X,A^	2.92 ± 0.50	2.30 ± 0.12 ^X,B^	3.79 ± 0.16 ^A^
	FRS	3.37 ± 0.03 ^Y,A^	2.66 ± 0.11 ^A^	2.00 ± 0.01 ^Y,B^	3.80 ± 0.01 ^C^
C6:0	CTL	2.20 ± 0.14	1.95 ± 0.03 ^x,A^	1.95 ± 0.08 ^Y^	2.20 ± 0.09 ^B^
	FRS	2.25 ± 0.21 ^A^	1.77 ± 0.07 ^y,A^	1.50 ± 0.07 ^X,B^	2.21 ± 0.19 ^A^
C8:0	CTL	1.10 ± 0.08	1.22 ± 0.19	1.10 ± 0.04 ^X^	1.22 ± 0.05
	FRS	1.25 ± 0.05 ^A^	0.89 ± 0.04 ^B^	0.88 ± 0.03 ^Y,B^	1.22 ± 0.18
C10:0	CTL	2.33 ± 0.11	2.31 ± 0.01 ^X^	2.19 ± 0.09 ^X^	2.32 ± 0.10
	FRS	2.12 ± 0.05 ^A^	1.40 ± 0.06 ^Y,B^	1.63 ± 0.02 ^Y,A^	2.33 ± 0.25 ^A^
C12:0	CTL	2.94 ± 0.05 ^X^	3.04 ± 0.04 ^X^	2.93 ± 0.12 ^X^	2.93 ± 0.12
	FRS	2.62 ± 0.04 ^Y,aA^	1.65 ± 0.07 ^Y,aB^	2.13 ± 0.18 ^Y,b^	2.82 ± 0.15 ^aA^
C14:0	CTL	13.80 ± 0.01 ^X,A^	14.74 ± 0.05 ^X,B^	14.50 ± 0.60 ^X^	14.14 ± 0.58
	FRS	11.84 ± 0.11 ^Y,A^	8.82 ± 0.36 ^Y,B^	11.08 ± 0.07 ^Y,C^	13.03 ± 0.21 ^D^
C14:1	CTL	0.92 ± 0.02 ^A^	1.06 ± 0.04 ^X,B^	1.07 ± 0.04 ^X,B^	1.07 ± 0.04 ^X,B^
	FRS	0.94 ± 0.01 ^X,A^	0.63 ± 0.03 ^Y,B^	0.78 ± 0.04 ^Y,C^	0.92 ± 0.02 ^Y,D^
C15:0	CTL	1.28 ± 0.04 ^X^	1.27 ± 0.04 ^X^	1.27 ± 0.05 ^x^	1.17 ± 0.05
	FRS	0.98 ± 0.02 ^Y,a^	0.88 ± 0.04 ^Y,b^	1.09 ± 0.06 ^y^	1.17 ± 0.08 ^b^
C16:0	CTL	36.16 ± 0.66 ^X^	35.86 ± 0.56 ^x^	36.96 ± 1.52	35.46 ± 1.46
	FRS	30.10 ± 0.07 ^Y,A^	32.70 ± 1.35 ^y^	34.88 ± 0.04 ^B^	35.10 ± 0.12 ^B^
C16:1	CTL	2.56 ± 0.06 ^X^	2.66 ± 0.06 ^X^	2.66 ± 0.11	2.44 ± 0.10
	FRS	2.16 ± 0.01 ^Y,A^	2.19 ± 0.09 ^Y,A^	2.50 ± 0.01 ^B^	2.67 ± 0.12 ^B^
C17:0	CTL	0.47 ± 0.01 ^X,A^	0.56 ± 0.06	0.56 ± 0.02 ^B^	0.47 ± 0.02 ^x,A^
	FRS	0.58 ± 0.02 ^Y,A^	0.59 ± 0.02 ^A^	0.58 ± 0.05 ^A^	0.57 ± 0.03 ^y^
C18:0	CTL	10.06 ± 0.09 ^X,A^	10.09 ± 0.19 ^X,A^	10.19 ± 0.42 ^X,a^	8.81 ± 0.36 ^bB^
	FRS	11.87 ± 0.25 ^Y,aA^	15.05 ± 0.62 ^Y,B^	12.62 ± 0.14 ^Y,Ab^	9.02 ± 0.12 ^C^
C18:1*n*-9 *trans*	CTL	1.45 ± 0.13 ^X,A^	1.44 ± 0.06 ^X,A^	0.99 ± 0.04 ^B^	1.54 ± 0.06 ^x,A^
	FRS	2.04 ± 0.10 ^Y,A^	2.54 ± 0.10 ^Y,B^	1.11 ± 0.23 ^aC^	1.72 ± 0.06 ^y,bC^
C18:1*n*-9 *cis*	CTL	17.67 ± 0.30 ^X^	17.75 ± 0.33 ^X^	17.68 ± 0.73 ^X^	18.84 ± 0.78
	FRS	22.95 ± 0.22 ^Y,A^	23.76 ± 0.98 ^Y,A^	23.53 ± 0.29 ^Y,A^	20.13 ± 0.12 ^B^
C18:2*n*-6 *cis*	CTL	1.04 ± 0.11 ^X,a^	1.40 ± 0.09 ^x,b^	1.22 ± 0.05 ^x^	1.22 ± 0.05 ^b^
	FRS	1.83 ± 0.04 ^Y,A^	1.66 ± 0.07 ^y,A^	1.64 ± 0.15 ^y^	1.32 ± 0.02 ^B^
C18:3*n*-3	CTL	0.31 ± 0.01 ^X,a^	0.30 ± 0.02	0.34 ± 0.01 ^b^	0.35 ± 0.01 ^x,b^
	FRS	0.42 ± 0.01 ^Y,A^	0.32 ± 0.01 ^Y,B^	0.31 ± 0.02 ^B^	0.31 ± 0.01 ^y,B^
CLA *cis*-9, *trans*-11	CTL	0.46 ± 0.05 ^X^	0.46 ± 0.01 ^X,A^	0.44 ± 0.02 ^X,A^	0.55 ± 0.02 ^B^
	FRS	0.71 ± 0.02 ^Y,aA^	0.84 ± 0.03 ^Y,B^	0.59 ± 0.02 ^Y,C^	0.58 ± 0.06 ^cB^

FRS, group of buffaloes fed with green forage; CTL, group of buffaloes fed without green forage. L; raw buffalo milk; T0, 1 d; MI, time of innovative method; MT, time of traditional method. FA, fatty acids. On each storage day, three samples by experimental group were analyzed. Statistical analysis was performed comparing experimental groups at each sampling time and within each experimental group along the ripening period. All data were presented as mean (m) ± standard error (se). Different superscript uppercase letters indicate a significant difference at *p* < 0.01. Different superscript lowercase letters indicate a significant difference at *p* < 0.05. ^A–D, a–c^ Mean values in the same row (same batch in different weeks) with different letters presented significant differences. ^X–Y, x–y^ Mean values in the same column (different samples on the same time/ripening time) with different letters presented significant differences.

**Table 8:** Microbiological (Log (cfu/g)) and physicochemical results of semi-hard buffalo cheeses.

		Raw Material	Semi-Finished Products		Ripened Cheeses	
Item		L	C	T0	MI	MT
TAB 30 °C	CTL	10.27 ± 0.41 ^A^	7.26 ± 0.06 ^X,aB^	6.86 ± 0.17 ^bB^	8.29 ± 0.30 ^bC^	7.90 ± 0.12 ^C^
	FRS	10.71 ± 0.17 ^A^	5.30 ± 0.21 ^Y,B^	7.14 ± 0.17 ^C^	8.17 ± 0.10 ^D^	8.14 ± 0.17 ^D^
TAB 7 °C	CTL	9.24 ± 0.17 ^A^	5.54 ± 0.27 ^X,B^	5.24 ± 0.30 ^X,aB^	6.30 ± 0.40 ^bB^	ni ^C^
	FRS	9.04 ± 0.48 ^A^	3.96 ± 0.08 ^Y,B^	3.96 ± 0.08 ^Y,B^	6.19 ± 0.20 ^C^	ni ^D^
Total Coliforms	CTL	7.44 ± 0.31 ^A^	5.66 ± 0.20 ^X,B^	4.24 ± 0.37 ^C^	2.10 ± 0.07 ^x,D^	3.66 ± 0.26 ^C^
	FRS	7.80 ± 0.12 ^A^	3.24 ± 0.37 ^Y,B^	4.77 ± 0.12 ^C^	1.56 ± 0.25 ^y,D^	3.44 ± 0.32 ^B^
Enterobacteriaceae	CTL	7.96 ± 0.24 ^A^	5.26 ± 0.27 ^X,B^	4.14 ± 0.25 ^C^	1.86 ± 0.18 ^D^	2.96 ± 0.09 ^E^
	FRS	7.74 ± 0.16 ^A^	3.26 ± 0.23 ^Y,aB^	4.24 ± 0.38 ^bB^	1.44 ± 0.32 ^C^	2.96 ± 0.29 ^aB^
*E. coli* glucorinidasi positive	CTL	ni ^A^	ni ^A^	ni ^A^	ni ^X,A^	2.00 ± 0.06 ^X,B^
	FRS	ni ^A^	ni ^A^	ni ^A^	1.44 ± 0.32 ^Y,B^	0.96 ± 0.09 ^Y,B^
*Enterococcus* spp.	CTL	9.55 ± 0.19 ^x,A^	5.71 ± 0.16 ^B^	5.60 ± 0.09 ^X,B^	7.28 ± 0.15 ^C^	7.24 ± 0.38 ^C^
	FRS	8.44 ± 0.40 ^y,aA^	5.04 ± 0.48 ^B^	4.26 ± 0.42 ^Y,B^	7.53 ± 0.04 ^bA^	7.46 ± 0.25 ^bA^
*Pseudomonas* spp.	CTL	7.78 ± 0.19 ^X,A^	4.91 ± 0.06 ^X,B^	3.74 ± 0.14 ^C^	1.96 ± 0.13 ^X,D^	ni ^E^
	FRS	6.74 ± 0.20 ^Y^	1.96 ± 0.08 ^Y^	3.90 ± 0.05	ni ^Y^	ni
Coagulase-positive staphylococci	CTL	7.23 ± 028 ^X,A^	5.78 ± 0.19 ^x,B^	3.34 ± 0.09 ^C^	3.34 ± 0.27 ^X,C^	ni ^D^
	FRS	6.53 ± 0.16 ^Y,A^	4.99 ± 0.34 ^y,B^	3.45 ± 0.19 ^C^	ni ^Y,D^	ni ^D^
*Lactobacillus* spp.	CTL	9.11 ± 0.44 ^aA^	5.79 ± 0.11 ^B^	5.54 ± 0.19 ^X,B^	8.11 ± 0.06 ^bA^	7.88 ± 0.20 ^bA^
	FRS	9.16 ± 0.15 ^A^	5.24 ± 0.30 ^B^	4.66 ± 0.12 ^Y,B^	7.95 ± 0.29 ^C^	7.98 ± 0.37 ^C^
Yeast	CTL	2.91 ± 0.06 ^A^	2.80 ± 0.25 ^X,A^	ni ^B^	ni ^B^	ni ^B^
	FRS	3.11 ± 0.39 ^A^	ni ^Y,B^	ni ^B^	ni ^B^	ni ^B^
Mold	CTL	4.30 ± 0.15 ^aA^	3.80 ± 0.33 ^X,A^	3.36 ± 0.17 ^X,B^	2.44 ± 0.78 ^X,bA^	ni ^C^
	FRS	3.96 ± 0.16 ^A^	1.96 ± 0.08 ^Y,B^	1.96 ± 0.35 ^Y,B^	ni ^Y,C^	ni ^C^
pH	CTL		5.44 ± 0.10 ^A^	5.40 ± 0.02 ^x,A^	5.31 ± 0.05 ^B^	5.31 ± 0.02 ^B^
	FRS		5.32 ± 0.02 ^A^	5.34 ± 0.01 ^y,aA^	5.23 ± 0.02 ^B^	5.29 ± 0.00 ^bA^
a_w_	CTL		0.973 ± 0.002 ^A^	0.969 ± 0.002 ^a^	0.920 ± 0.031	0.898 ± 0.026 ^bB^
	FRS		0.976 ± 0.002 ^a^	0.977 ± 0.006 ^a^	0.940 ± 0.018	0.929 ± 0.018 ^b^

FRS, group of buffaloes fed with green forage; CTL, group of buffaloes fed without green forage. L, raw buffalo milk; C, curd; T0, 1 d; MI, time of innovative method; MT, time of traditional method. ni: not isolated. On each storage day, three samples by experimental group were analyzed. Statistical analysis was performed comparing experimental groups at each sampling time and within each experimental group along the ripening period. All data were presented as mean (m) ± standard error (se). Different superscript uppercase letters indicate a significant difference at *p* < 0.01. Different superscript lowercase letters indicate a significant difference at *p* < 0.05. ^A–E, a–b^ Mean values in the same row (same batch in different weeks) with different letters presented significant differences. ^X–Y, x–y^ Mean values in the same column (different samples on the same time/ripening time) with different letters presented significant differences.

**Table 9:** Microbiological (Log (cfu/g)) and physicochemical results of dry ricotta buffalo cheeses.

		Raw Material	Semi-Finished Product	Ripened Cheeses	
Item		L	T0	MI	MT
TAB 30 °C	CTL	8.36 ± 0.16 ^A^	3.50 ± 0.47 ^B^	8.11 ± 0.25 ^A^	7.26 ± 0.14 ^X,C^
	FRS	7.68 ± 0.37 ^A^	4.38 ± 0.22 ^B^	8.01 ± 0.09 ^A^	6.44 ± 0.20 ^Y,C^
TAB 7 °C	CTL	8.42 ± 0.19 ^X,A^	3.21 ± 0.33 ^x,B^	7.71 ± 0.15 ^C^	7.46 ± 0.20 ^C^
	FRS	7.70 ± 0.11 ^Y,aA^	4.10 ± 0.08 ^y,B^	7.41 ± 0.18 ^A^	7.44 ± 0.05 ^Ba^
Total Coliforms	CTL	7.70 ± 0.08 ^x,A^	1.74 ± 0.15 ^X,B^	7.86 ± 0.08 ^X,A^	8.04 ± 0.37 ^X,A^
	FRS	7.01 ± 0.31 ^y,A^	4.14 ± 0.03 ^Y,B^	7.21 ± 0.14 ^Y,A^	6.69 ± 0.12 ^Y,A^
Enterobacteriaceae	CTL	6.96 ± 0.09 ^x,A^	1.66 ± 0.19 ^X,B^	6.82 ± 0.18 ^A^	6.41 ± 0.35 ^A^
	FRS	7.26 ± 0.10 ^y,aA^	3.26 ± 0.43 ^Y,B^	6.79 ± 0.17 ^bA^	6.04 ± 0.75 ^A^
*E. coli* glucorinidasi positive	CTL	4.67 ± 0.37 ^A^	ni ^B^	ni ^X,B^	ni ^B^
	FRS	3.86 ± 0.21 ^A^	ni ^B^	1.26 ± 0.14 ^Y,C^	ni ^B^
*Enterococcus* spp.	CTL	8.26 ± 0.08 ^x,A^	2.80 ± 0.17 ^B^	7.53 ± 0.21 ^C^	6.26 ± 0.27 ^D^
	FRS	7.59 ± 0.23 ^y,A^	3.45 ± 0.31 ^B^	7.07 ± 0.52 ^aA^	5.96 ± 0.12 ^bC^
*Pseudomonas* spp.	CTL	7.21 ± 0.08 ^x,aA^	ni ^X,B^	5.84 ± 0.50 ^X,bAC^	6.86 ± 0.09 ^C^
	FRS	6.74 ± 0.16 ^y,aA^	4.24 ± 0.14 ^Y,B^	7.53 ± 0.25 ^Y,bA^	6.86 ± 0.12 ^aA^
Coagulase-positive staphylococci	CTL	6.35 ± 0.14 ^X,A^	2.10 ± 0.27 ^X,B^	4.71 ± 0.03 ^C^	4.44 ± 0.13 ^C^
	FRS	5.45 ± 0.12 ^Y,A^	3.45 ± 0.10 ^Y,B^	4.99 ± 0.11 ^A^	4.44 ± 0.10 ^C^
*Lactobacillus* spp. 30	CTL	8.52 ± 0.25 ^x,A^	3.00 ± 0.23 ^X,B^	7.70 ± 0.11 ^C^	7.26 ± 0.05 ^X,D^
	FRS	7.64 ± 0.20 ^y,A^	4.29 ± 0.17 ^Y,B^	7.46 ± 0.30 ^A^	6.44 ± 0.17 ^Y.C^
Yeast	CTL	3.44 ± 0.12 ^A^	ni ^B^	4.74 ± 0.22 ^x,C^	3.64 ± 0.21 ^A^
	FRS	3.50 ± 0.15 ^A^	ni ^B^	3.96 ± 0.28 ^y,A^	4.66 ± 0.65 ^A^
Mold	CTL	4.91 ± 0.09 ^x,A^	2.91 ± 0.11 ^X,B^	7.00 ± 0.07 ^C^	6.91 ± 0.27 ^C^
	FRS	4.64 ± 0.05 ^y,A^	3.90 ± 0.19 ^Y,B^	6.69 ± 0.18 ^aC^	7.19 ± 0.09 ^bC^
pH	CTL		6.42 ± 0.04 ^A^	5.82 ± 0.03 ^B^	6.01 ± 0.02 ^X,B^
	FRS		6.43 ± 0.02 ^A^	5.70 ± 0.16 ^B^	5.63 ± 0.07 ^Y,B^
a_w_	CTL		0.978 ± 0.004 ^A^	0.964 ± 0.009 ^A^	0.820 ± 0.028 ^B^
	FRS		0.968 ± 0.010 ^A^	0.952 ± 0.012 ^A^	0.780 ± 0.021 ^B^

FRS, group of buffaloes fed with green forage; CTL, group of buffaloes fed without green forage. L, raw buffalo milk; T0, 1 d; MI, time of innovative method; MT, time of traditional method. ni: not isolated. On each storage day, three samples by experimental group were analyzed. Statistical analysis was performed comparing experimental groups at each sampling time and within each experimental group along the ripening period. All data were presented as mean (m) ± standard error (se). Different superscript uppercase letters indicate a significant difference at *p* < 0.01. Different superscript lowercase letters indicate a significant difference at *p* < 0.05. ^A–D, a–b^ Mean values in the same row (same batch in different weeks) with different letters presented significant differences. ^X–Y, x–y^ Mean values in the same column (different samples on the same time/ripening time) with different letters presented significant differences.

## Data Availability

Data available within the article. The authors confirm that the data supporting the findings of this study are available within the article.

## Supplementary Materials

The following supporting information can be downloaded at: https://www.mdpi.com/article/10.3390/foods12040704/s1, Figure S1: industrial ripening plant used to ripen cheeses; Table S1: diets composition (kg) of buffaloes fed with (FRS) or without (CTL) green feed inclusion; Table S2: chemical composition of buffalo milks used to produce the cheeses; Table S3: effects of feeding system and ripening time on SCFA, MCFA, LCFA; SFA, MUFA and PUFA composition; n-3, n-6 CLAs composition in semi-hard cheeses; Table S4: effects of feeding system and ripening time on SCFA, MCFA, LCFA; SFA, MUFA and PUFA composition; n-3, n-6 CLAs composition in dry ricotta cheeses
