# Supplementary material for: Role of Feeding and Novel Ripening System to Enhance the Quality and Production Sustainability of Curd Buffalo Cheeses
Source: Foods. 2023 Feb 6;12(4):704. doi: 10.3390/foods12040704 (PMC9956207; doi:10.3390/foods12040704)
Supplement: Supplementary file 1 [file foods-12-00704-s001.zip › foods-2150282-supplementary.pdf]

FOODS-2150282
Role of feeding and novel ripening system to enhance the quality and production sustainability of curd buffalo cheeses

SUPPLEMENTARY DATA

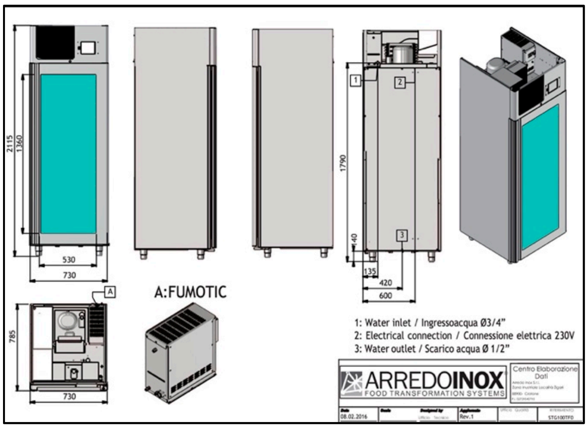

Figure S1. Industrial ripening plant used to ripen cheeses. All dimensions are in mm.

Table S1. Diets composition (kg) of buffaloes fed with (FRS) or without (CTL) green feed inclusion.

Feed (Kg)	Diet	
	CTL	FRS
Concentrate	5.50	2.50
Alfalfa	4.50	2.60
Straw	1.20	1.20
Silomais	23.00	18.00
Green ryegrass	-	25.00
Hydrogenated fats	-	0.30
Calcium carbonate	-	0.05
TOTAL	34.20	49.65

Table S2. Chemical composition of buffalo milks used to produce the cheeses

		Raw milk (L)	
Item		Semi-hard cheese	Dry ricotta cheese
Fat,	CTL	7.20±0.23^{X}	7.80±0.17^{x}
%	FRS	9.10±0.12^{Y}	8.60±0.17^{y}
Protein,	CTL	4.70±0.06	4.90±0.06
%	FRS	4.40±0.12	4.60±0.12
Solid not-fat,	CTL	10.15±0.20	10.02±0.03
%	FRS	9.97±0.11	10.00±0.07
Lactose,	CTL	4.76±0.05	4.64±0.17
%	FRS	4.73±0.02	4.60±0.20

FRS, group of buffaloes fed with green forage; CTL, group of buffaloes fed without green forage. Statistical analysis was performed comparing experimental groups. All data were presented as mean (m) ± standard error (se). Different superscript uppercase letters indicate a significant difference at $p < 0.01$. Different superscript lowercase letters indicate a significant difference at $p < 0.05$.
$^{x\text{-}y}$ Mean values in the same column with different letters presented significant differences.

Table S3. Effects of feeding system and ripening time on SCFA, MCFA, LCFA; SFA, MUFA and PUFA composition; n-3, n-6 CLAs composition in semi-hard cheeses

		Raw material	Semi-finished products		Ripened cheeses	
**Item**		**L**	**C**	**T0**	**MI**	**MT**
SCFA	CTL	7.05±0.70^{x,A}	11.14±0.37^{X,B}	9.81±0.23^{C}	8.06±0.13^{X,aA}	7.54±0.14^{bA}
	FRS	8.95±0.15^{y,aA}	9.59±0.23^{Y,bA}	10.09±0.22^{C}	6.46±0.06 ^{Y,D}	7.87±0.14^{E}
MCFA	CTL	59.46±1.14^{a}	57.39±1.89	56.93±1.30	56.19±0.93^{X,b}	55.72±1,05^{X,b}
	FRS	60.03±1.11^{aA}	56.6±1.34^{A}	56.35±1.29^{bA}	47.15±0.78^{Y,B}	48.61±0.84^{Y,B}
LCFA	CTL	34.30±0.64^{aA}	32.69±1.07^{aA}	34.04±0.78^{aA}	36.20±0.60^{X,b}	37.04±0.70^{X,B}
	FRS	32.76±0.69^{A}	34.47±0.82^{A}	34.24±0.78^{A}	44.81±0.74^{Y,B}	43.60±0.76^{Y,B}
SFA	CTL	73.58±1.32^{a}	72.88±2.40	71.55±1.64	70.26±1.16^{X}	69.20±1.30^{b}
	FRS	74.40±1.46^{A}	72.45±1.71^{aA}	72.52±1.66^{aA}	65.50±1.09^{Y,B}	67.93±1.18^{bB}
MUFA	CTL	26.11±0.50	24.31±0.80^{aA}	25.48±0.58^{ab}	26.65±0.44^{X,bc}	27.60±0.52^{cB}
	FRS	24.71±0.56^{A}	25.00±0.59^{A}	25.06±0.57^{A}	30.50±0.51^{Y,aB}	28.64±0.50^{bB}
PUFA	CTL	2.69±0.08^{aA}	3.11±0.10^{X,B}	2.99±0.07^{X,b}	3.10±0.05^{X,B}	3.24±0.06^{X,aB}
	FRS	2.84±0.06^{A}	2.56±0.06^{Y,B}	2.43±0.06^{Y,b}	4.01±0.07^{Y,aC}	3.81±0.07^{Y,bC}
CLA	CTL	0.69±0.02^{X,A}	0.58±0.02^{B}	0.58±0.01^{B}	0.59±0.01^{X,B}	0.58±0.01^{X,B}
	FRS	0.48±0.01^{Y,A}	0.57±0.01^{B}	0.58±0.01^{B}	0.72±0.01^{Y,C}	0.72±0.01^{Y,C}
n-3	CTL	0.31±0.01^{X,A}	0.31±0.01^{X,A}	0.31±0.01^{A}	0.31±0,01^{X,A}	0.36±0.01^{X,B}
	FRS	0.43±0.01^{Y,A}	0.39±0.01^{Y,B}	0.31±0.01^{C}	0.51±0.01^{Y,D}	0.61±0.01^{Y,E}
n-6	CTL	1.74±0.03^{X,A}	2.02±0.07^{X,B}	1.99±0.05^{X,B}	2.01±0.03^{B}	1.81±0.03^{X}
	FRS	1.93±0.04^{Y}	1.59±0.04^{Y}	1.43±0.03^{Y}	2.11±0.04	2.01±0.04^{Y}
n-6/n-3	CTL	5.69±0.14^{X,A}	6.51±0.21^{X,B}	6.49±0.15^{X,B}	6.49±0.11^{X,B}	5.08±0.10^{X,C}
	FRS	4.47±0.05^{Y,A}	4.05±0.10^{Y,B}	4.63±0.11^{Y,A}	4.16±0.07^{Y,B}	3.27±0.06^{Y,C}

FRS, group of buffaloes fed with green forage; CTL, group of buffaloes fed without green forage. L, raw buffalo milk; C, curd; T0, 1 d; MI, time of innovative method; MT, time of traditional method. SCFA, Short-chain fatty acids (C4 to C10); MCFA, Medium-Chain Fatty Acids (C12- C16); LCFA, Long-Chain Fatty Acids (C18:0 - C18:3); MUFA, monounsaturated fatty acids; PUFA, polyunsaturated fatty acids; SFA, saturated fatty acids. In each storage day, three samples by experimental group were analysed. Statistical analysis was performed comparing experimental groups at each sampling time and within each experimental group along the ripening period. All data were presented as mean (m) ± standard error (se). Different superscript uppercase letters indicate a significant difference at $p < 0.01$. Different superscript lowercase letters indicate a significant difference at $p < 0.05$.

$^{a-d}$ Mean values in the same row (same batch in different weeks) with different letters presented significant differences.

$^{x-y}$ Mean values in the same column (different samples on the same time/ripening time) with different letters presented significant differences.

Table S4. Effects of feeding system and ripening time on SCFA, MCFA, LCFA; SFA, MUFA and PUFA composition; n-3, n-6 CLAs composition in dry ricotta cheeses

		Raw material	Semi-finished product	Ripened cheeses	
**Item**		**L**	**T0**	**MI**	**MT**
SCFA	CTL	9.55±0.40^{aA}	11.45±0.37^{X,A}	11.09±0.46^{X,B}	12.46±0.51^{B}
	FRS	9.00±0.33^{A}	8.38±0.34^{Y,A}	8.14±0.23^{Y,A}	12.37±0.46^{B}
MCFA	CTL	59.98±0.70^{X}	58.64±0.67^{X}	59.39±2.45^{x}	57.22±2.36
	FRS	50.77±0.18^{Y,A}	46.88±1.93^{Y,aA}	52.46±0.03^{y,bB}	55.71±0.13^{C}
LCFA	CTL	30.53±0.62^{X}	31.55±0.75^{X}	30.98±1.28^{X}	31.23±1.29
	FRS	39.10±0.59^{Y,bA}	43.91±1.81^{Y,aA}	39.79±0.16^{Y,bA}	33.06±0.30^{B}
SFA	CTL	74.79±0.74^{X}	74.26±0.30^{x}	74.99±3.09	72.90±3.00
	FRS	67.83±0.21^{Y,A}	66.63±2.74^{y}	68.59±0.82^{A}	71.46±0.33^{B}
MUFA	CTL	22.97±0.25^{X}	23.20±0.12^{X}	22.68±0.93^{X}	24.17±1.00
	FRS	28.45±0.10^{Y,A}	29.42±1.21^{Y,a}	28.22±0.99^{Y,a}	25.73±0.51^{bB}
PUFA	CTL	2.40±0.51	2.54±0.27^{X}	2.36±0.10^{X,A}	2.96±0.12^{X,B}
	FRS	3.60±0.37^{ab}	3.76±0.15^{Y,aA}	3.19±0.15^{Y,bA}	2.60±0.03^{y,abB}
CLA	CTL	0.46±0.01^{X,A}	0.46±0.02 ^{X,A}	0.44±0.02^{X,A}	0.55±0.02^{B}
	FRS	0.82±0.03^{Y,aA}	0.95±0.04^{Y,bA}	0.59±0.02^{Y,B}	0.58±0.02^{B}
n-3	CTL	0.39±0.02^{X,A}	0.30±0.01^{B}	0.34±0.01	0.35±0.01
	FRS	0.50±0.03^{Y,A}	0.32±0.01^{B}	0.41±0.06	0.31±0.05^{B}
n-6	CTL	1.55±0.03^{X,A}	1.78±0.05^{X,B}	1.49±0.06^{X,A}	1.98±0.08^{Y,B}
	FRS	2.28±0.03^{Y,A}	2.32±0.10^{Y,A}	2.18±0.08^{Y,A}	1.72±0.03^{Y,B}
n-6/n-3	CTL	4.01±0.26^{A}	5.84±0.02^{X,B}	4.33±0.18^{X,A}	5.72±0.24^{B}
	FRS	4.57±0.19^{A}	7.31±0.30^{Y,B}	5.31±0.14^{Y,C}	5.61±0.16^{B}

FRS, group of buffaloes fed with green forage; CTL, group of buffaloes fed without green forage. L, raw buffalo milk; C, curd; T0, 1 d; MI, time of innovative method; MT, time of traditional method. SCFA, Short-chain fatty acids (C4 to C10); MCFA, Medium-Chain Fatty Acids (C12- C16); LCFA, Long-Chain Fatty Acids (C18:0 - C18:3); MUFA, monounsaturated fatty acids; PUFA, polyunsaturated fatty acids; SFA, saturated fatty acids. In each storage day, three samples by experimental group were analysed. Statistical analysis was performed comparing experimental groups at each sampling time and within each experimental group along the ripening period. All data were presented as mean (m) ± standard error (se). Different superscript uppercase letters indicate a significant difference at $p < 0.01$. Different superscript lowercase letters indicate a significant difference at $p < 0.05$.

$^{a-c}$ Mean values in the same row (same batch in different weeks) with different letters presented significant differences.

$^{x-y}$ Mean values in the same column (different samples on the same time/ripening time) with different letters presented significant differences.
